# Norditerpenoids from *Flickingeria fimbriata* and Their Inhibitory Activities on Nitric Oxide and Tumor Necrosis Factor-α Production in Mouse Macrophages

**DOI:** 10.3390/molecules19055863

**Published:** 2014-05-06

**Authors:** Jin-Long Chen, Wen-Jun Zhong, Gui-Hua Tang, Jing Li, Zhi-Min Zhao, De-Po Yang, Lin Jiang

**Affiliations:** School of Pharmaceutical Sciences, Sun Yat-sen University, Guangzhou 510006, Guangdong, China; E-Mails: jlchen5858@163.com (J.-L.C.); clockzwj@163.com (W.-J.Z.); tanggh5@mail.sysu.edu.cn (G.-H.T.); lijing45@mail2.sysu.edu.cn (J.L.); zhaozhimin1978@hotmail.com (Z.-M.Z.)

**Keywords:** norditerpenoid, TDDFT, exiton chirality method, NO, TNF-α

## Abstract

Bioassay-guided fractionation of the ethanolic extract of the leaves of *Flickingeria flimbriata* led to the isolation of two new degraded diterpenoids **1** and **2**, a new *ent*-pimarane type diterpenoid **3**, and four known steroids **4**–**7**. The structures of **1**–**3** were elucidated by spectroscopic analysis, and their absolute configurations were determined by chemical methods, TDDFT quantum chemical calculations of ECD spectra, and CD exiton chirality method. Compounds **1** and **2**, named flickinflimilins A and B, possess a rare 15,16-dinor-*ent*-pimarane skeleton. Compounds **1**–**7** were screened for the inhibitory activity against lipopolysaccharide (LPS)-induced NO and TNF-α production in RAW264.7 cells. Compounds **1**–**3** exhibited potent inhibitory activities, with IC_50_ values of less than 10 µM.

## 1. Introduction

*Flickingeria fimbriata* (Bl.) Hawkes (Orchidaceae), is widely used as a substitute of the precious and scarce Traditional Chinese Medicine *Dendrobium candidum*, for treatment of pneumonia, tuberculosis, asthma, and pleurisy [1]. Phytochemical investigations of *Flickingeria* species have revealed the occurrence of diterpenes [2,3,4,5,6], phenanthrenes [4], steroids [5], and bibenzyls [6]. These natural products are reported possessing P-glycoprotein inhibition [2], anti-inflammation [7,8,9], and antimutagenic activities [10].

**Figure 1:**
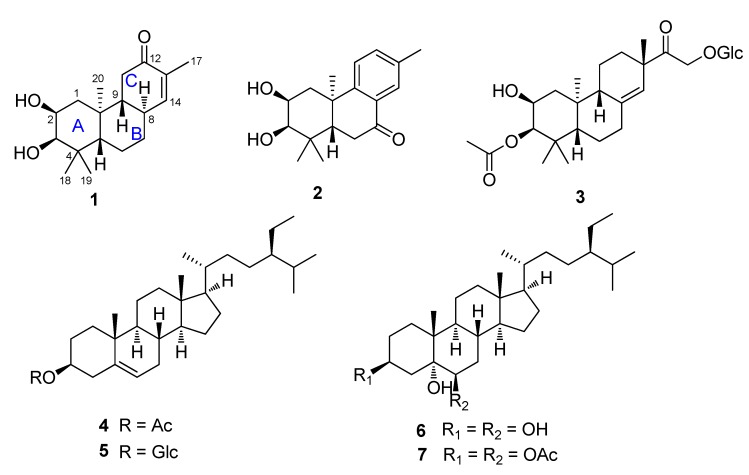
The structures of compounds **1**–**7**.

In our continuing search for anti-inflammatory metabolites from medical plants, a fraction of the EtOAc extract partitioned from the ethanolic extract of leaves of *F*. *fimbriata* showed an inhibitory activity of 42.2% against NO production and 40.1% against TNF-α production, respectively, at a concentration of 10 µM. Column chromatographic separations led to the isolation of three new diterpenoids **1**–**3** together with four known steroids **4**–**7** (Figure 1). Flickinflimilins A and B (compounds **1**,**2**) represent an unusual group of 15,16-dinor-*ent*-pimarane. The isolated compounds **1**–**7** were screened for their inhibitory activity against lipopolysaccharide (LPS)-induced NO and TNF-α production in RAW264.7 cells. The results showed that compounds **1**–**3** exhibited potent inhibitory activities with the IC_50_ values less than 10 µM. Herein, the details of the isolation, structural elucidation, and inhibitory activities of these compounds are described.

## 2. Results and Discussion

The air-dried powder of the leaves of *F*. *fimbriata* was extracted with 95% EtOH at room temperature to give a crude extract, which was then suspended in H_2_O and successively partitioned with petroleum ether, EtOAc, and *n*-BuOH, respectively. Various column chromatographic separations of the petroleum ether, EtOAc extract afforded compounds **1**–**7**.

**Figure 2:**
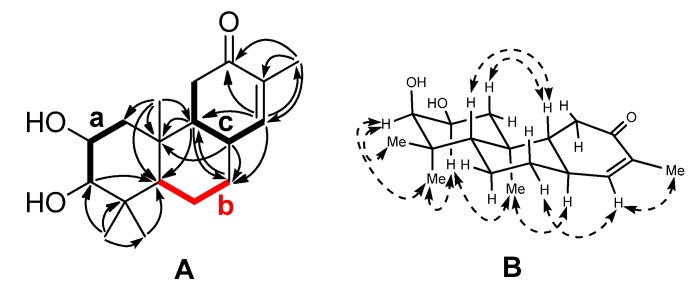
(**A**) Selected ^1^H-^1^H COSY (▬) and HMBC (→) correlations of **1**; (**B**) Selected NOESY correlations of **1** (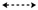).

Compound **1** was obtained as a colorless oil with the molecular formula C_18_H_28_O_3_ as established by an *m*/*z* of 315.1943 [M + Na]^+^ (calcd for C_18_H_28_O_3_Na, 315.1936) from HRESIMS. The ^1^H-NMR spectrum of **1** showed four tertiary methyls [at δ_H_ 2.27, 1.00, 0.94, and 0.87 (each 3H, s)], two oxygenated methines [at δ_H_ 4.05 (ddd, *J* = 2.8, 4.4, and 11.8 Hz, H-2) and 3.45 (d, *J* = 2.8 Hz, H-3)], and an olefinic proton [at δ_H_ 6.65 (t, *J* = 1.6 Hz, H-14)]. The ^13^C-NMR spectrum in combination with DEPT experiments showed 18 carbon resonances, including four quaternary carbons (one conjugated carbonyl and one olefinic), six methines (two oxygenated and one olefinic), four methylenes, and four quaternary methyls. The double bond and the carboxyl group accounted for two out of the five degrees of unsaturation, and the leftover double-bond equivalents required a tricyclic nature of **1**. The gross structure was constructed by two-dimensional (2D) NMR analysis. Three fragments (Figure 2), **a** (C-1 to C-3), **b** (C-5 to C-7), and **c** (C-14, C-8, C-9, and C-11) were established by the correlations observed in the ^1^H-^1^H COSY spectrum of **1**. The connectivity of these fragments, quaternary carbons and other substitutes was accomplished mainly by analysis of the HMBC spectrum (Figure 2). Fragment **a**, C-10 bearing the angular methyl group (C-20), the C-5 methine, and the *gem*-dimethyl group could construct ring A by the HMBC correlations from H_3_-20 to C-1, C-5, and C-10 and from H_3_-18/19 to C-3, C-4, and C-5. The HMBC correlations of H-9 to C-5, C-7, and C-10, H-7 to C-9, and H-8 to C-7, C-9 and C-10 supported that ring B was constructed by C-8–C-10 and fragment **b**, and fused with ring A via C-5 and C-10. Fragment **c** and the α,β-unsaturated ketone group provide ring C by the HMBC correlations from H-14 to C-8, C-9, C-12, and C-13. The planar structure of **1** thus emerged from the above spectra analysis.

The relative configuration of **1** was determined by analysis of the NOESY correlations (Figure 2) and coupling constants. The NOESY spectrum showed cross peaks between the proton pairs H-2/H_3_-19, H-2/H_3_-20, indicating that these protons were cofacial and axial oriented, which were arbitrarily assigned as α orientation. Accordingly, a chair conformation for ring A was assigned. Based on the small coupling constant (*J* = 2.6 Hz) between H-2 and H-3, the OH-3 was assigned as β orientation. The β assignment of coplanar protons H-5 and H-9 were deduced from the NOE correlations of H-1β/H-5, H-5/H-7β, and H-5/H-9.

**Figure 3:**
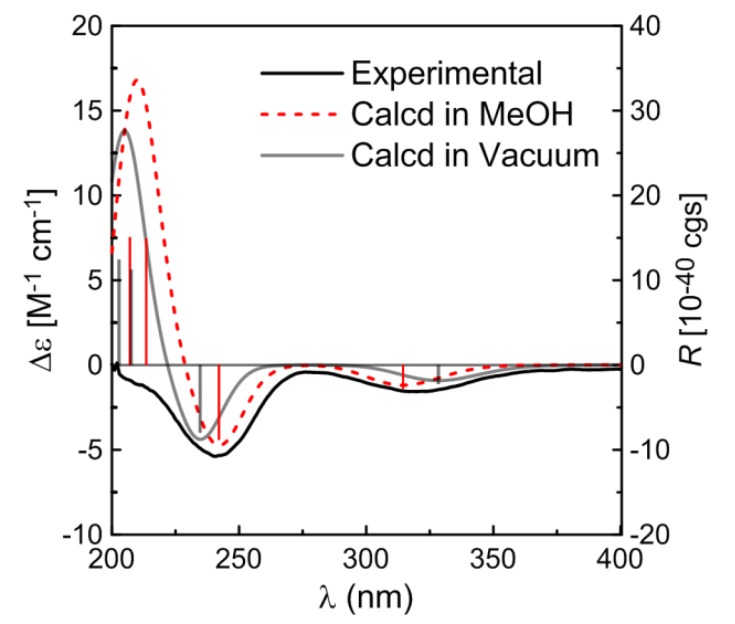
Calculated CD spectra of compound **1** in MeOH solution (red) and in vacuum (gray) and comparison between the calculated and experimental CD (black) spectra. Vertical bars represent rotational strengths *R*. σ = 0.20 eV.

The absolute configuration of **1** was established by comparing the experimental ECD spectrum with the calculated data. The TDDFT calculations were performed using the B3LYP functional and TZVP basis set. Then conformer of **1** were subjected to TDDFT calculations for solution CD in MeOH and also calculated for the gas phase CD. The simulated CD spectra (Figure 3) exhibited negative Cotton effects (CEs) around 320 nm and 240 nm and a positive CE around 210 nm, all generally consistent with the experimental spectrum, and the weighted spectra in both the gas phase and MeOH solution (Figure 3) provided the excellent fit with the experimental data, giving a firm support to the determined absolute configuration of **1**.

Compound **2** was obtained as a white amorphous powder, and its molecular formula was determined to be C_18_H_24_O_3_ (seven degrees of unsaturation) from the quasi-molecular ion peak at *m*/*z* 311.1987 [M + Na]^+^ (calcd for C_18_H_24_O_3_Na, 311.1989) in the HRESIMS spectrum. The IR absorption bands at 3440, 1708, 1600, 1500, and 1476 cm^−1^ indicated the presence of hydroxyl, carbonyl, and aromatic groups. The ^1^H-NMR spectrum of **2** (Table 1) showed the signals of four tertiary methyl groups [at δ_H_ 2.34, 1.25, 1.19, and 1.03 (each 3H, s)], two oxygenated methines [at δ_H_ 4.26 (ddd, *J* = 2.6, 4.5, and 11.5 Hz, H-2) and 3.61 (d, *J* = 2.6 Hz, H-3)], and three aromatic protons [at δ_H_ 7.80 (d, *J* = 1.3 Hz, H-14), 7.34 (dd, *J* = 1.3, 8.0 Hz, H-12), and 7.27 (d, *J* = 8.0 Hz, H-11)]. The ^13^C-NMR spectrum in combination with DEPT experiments showed 18 carbon resonances, including six quaternary carbons (one conjugated carbonyl and three olefinic carbons), six methines (two oxygenated and three olefinic carbons), two methylenes, and four methyls. The double bonds and ketone group accounted for four out of the seven degrees of unsaturation, the remaining three double-bond equivalents required **2** to be tricyclic. The aforementioned data resembled those of compound **1**. The major structural differences were due to the location of the conjugated ketone group and C-ring aromatized in **2**. The structure of **2** was further demonstrated by HMBC and NOESY spectra (See supplementary materials Figure S27). Compound **2** represents the first C-ring aromatized dinor-*ent*-pimarane reported hitherto.

**Figure 4:**
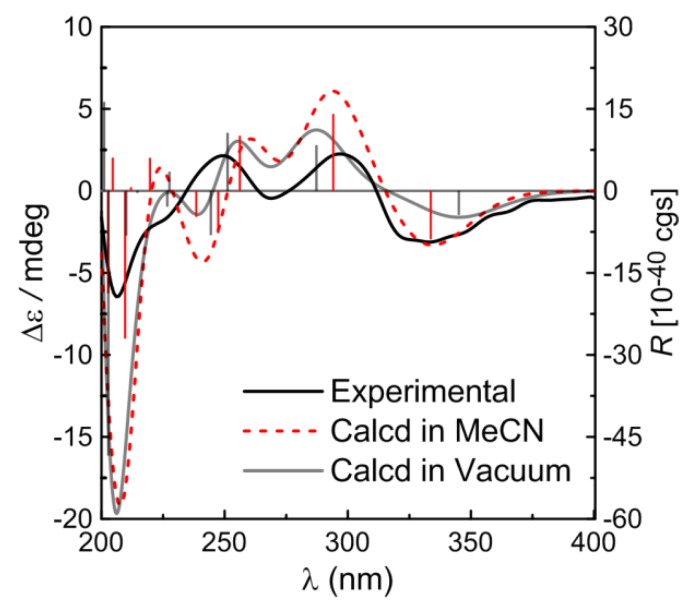
Calculated CD spectra of compound **2** in MeCN solution (red) and in vacuum (gray) and comparison between the calculated and experimental CD (black) spectra. Vertical bars represent rotational strengths *R*. σ = 0.24 eV.

The absolute configuration of compound **2** was postulated on the basis of comparison between the experimental ECD spectrum and the calculated data. As can be seen in Figure 4, the Boltzmann weighted CD spectra in both the gas phase and MeCN solution, in particular the solution spectrum, are in good agreement with the experimental spectrum.

Compound **3**, a colorless oil, had a molecular formula of C_28_H_44_O_10_ as determined by HRESIMS *m*/*z* 585.2929 [M + HCOO]^−^ (calcd for C_2__9_H_4__5_O_1__2_, 585.2923). Closely inspection of the ^1^H and ^13^C-NMR spectra indicated compound **3** was a diterpenoid glycoside. Comparison of the NMR data (Table 1) and molecular formula of **3** with those of ephemeranthoside [4] demonstrated that **3** had an additional acetyl group. [δ_H_2.10 s (3H); δ_C_ 21.3 and 173.0], which was located at C-3 from analysis of the HMBC spectrum. The relative configuration of 3 was established by analysis of the NOESY spectrum.

**Table 1:** ^1^H-NMR (400 MHz) and ^13^C-NMR (100 MHz) data for compounds **1**–**3** (δ in ppm).

No.	1 ^a^		2 ^b^		3 ^a^	
	δ_H_ (*J* in Hz)	δ_C_, type	δ_H_ (*J* in Hz)	δ_C_, type	δ_H_ (*J* in Hz)	δ_C_, type
1α	1.54, m	40.4, CH_2_	2.31, m	40.3, CH_2_	1.56, m	41.3, CH_2_
1β	1.44, t (11.8)		1.99, m		1.53, m	
2	4.05, ddd (2.8, 4.4, 11.8)	66.3, CH	4.26, ddd (2.6, 4.5, 11.5)	66.2, CH	4.00, ddd (2.6, 4.7, 11.7)	65.9, CH
3	3.45, d (2.8)	79.2, CH	3.61, d (2.6)	78.8, CH	4.91, d (2.6)	81.5, CH
4		38.2, C		38.8, C		39.9, C
5	1.37, m	47.6, CH	2.29, m	42.8, CH	1.44, m	49.9, CH
6α	1.58, m	21.7, CH_2_	2.64, d (1.4)	36.0, CH_2_	1.42, m	22.6, CH_2_
6β	1.37, m		2.62, m		1.57, m	
7α	2.13, m	30.2, CH_2_		199.4, C	2.12, m	36.5, CH_2_
7β	2.07, m				2.42, d (14.2)	
8	2.49, t (12.0)	45.8, CH		131.1, C		142.9, C
9	1.59, m	61.3, CH		153.7, C	1.92, t (9.1)	52.0, CH
10		37.1, C		39.2, C		40.5, C
11α	2.37, dd (3.8, 11.6)	29.2, CH_2_	7.27, d (8.0)	124.2, C	1.65, m	21.3, CH_2_
11β	2.15 br, s				1.24, m	
12α		197.4, C	7.34, dd (1.3, 8.0)	135.0, C	1.10, m	33.5, CH_2_
12β					2.31, d	
13		145.3, C		135.8, C		48.7, C
14α	6.65, t (1.6)	148.0, CH	7.80, d (1.3)	127.7, C	5.54, s	125.5, CH
14β						
15						213.5, C
16a					4.49, d (18.4)	72.4, CH_2_
16b					4.88, d (18.4)	
17	2.27, s	26.1, CH_3_	2.34, s	20.6, CH_3_	1.14, s	27.5, CH_3_
18	0.87, s	21.3, CH_3_	1.03, s	21.5, CH_3_	0.89, s	28.5, CH_3_
19	1.00, s	28.7, CH_3_	1.19, s	28.4, CH_3_	0.98, s	22.4, CH_3_
20	0.94, s	16.0, CH_3_	1.25, s	24.3, CH_3_	0.77, s	16.0, CH_3_
CH_3_CO					2.10, s	21.3, CH_3_
CH_3_CO						173.0, C
1’					4.24, d (7.6)	104.2, CH
2’					3.16, dd (7.6, 9.2)	75.0, CH
3’					3.27, dd (2.7, 5.0)	78.2, CH
4’					3.28, d (8.8)	71.5, CH
5’					3.24, dd (2.7, 5.0)	77.6, CH
6’a					3.87, dd (3.6, 12.0)	72.4, CH_2_
6’b					3.64, dd (3.6, 12.0)	

^a^ Measured in CDCl_3_; ^b^ Measured in Pyridine.

**Scheme 1:**
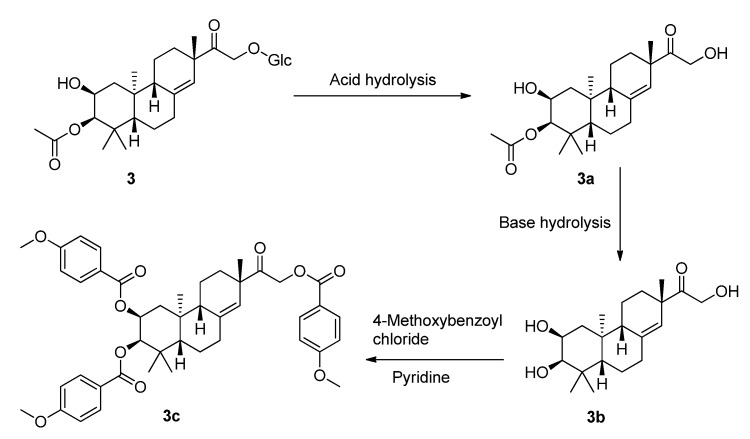
The chemical transformation from **3** to **3c**.

**Figure 5:**
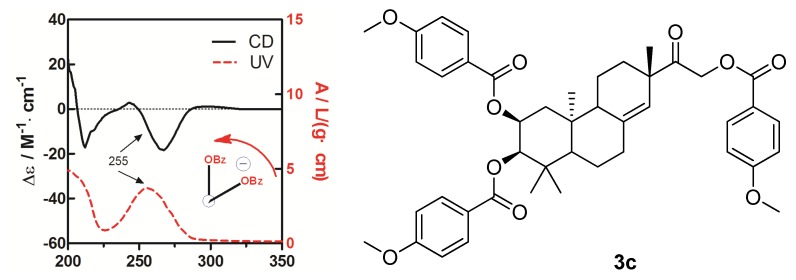
CD and UV spectra of compound **3c** in MeOH. The arrow denotes the electric transition dipole of the chromophores.

To determine the absolute configuration of compound **3**, the chemical transformation from **3** to **3c** was performed (as shown in Scheme 1), then the exciton chirality method was applied on 2,3,16-tri-*p*-methoxybenzoate derivative (**3c**). The negative chirality resulting from the exciton coupling between the two chromophores of *p*-methoxybenzoate at 267 nm (∆ε −18.41, π-π* transition) and 243 nm (∆ε +2.85, π-π* transition) indicated that the transition dipole moments of the two chromophores were oriented in a counterclockwise manner (Figure 5) [11]. Thus, the absolute configurations of C-2 and C-3 of **3c** were determined to be 2*S* and 3*R*, respectively. The absolute configuration of the β-glucose was identified to be d-configuration by HPLC analysis. Therefore, compound **3** was established as 2,16-dihydroxyl-15-keto-2-acetoxy-*ent*-pimar-8(14)-ene-16-*O*-β-d-glucopyranoside.

The known compounds sitost-5-en-3β-ol acetate (**4**) [12], β-sitosterol-3-*O*-β-d-glucopyranoside (**5**) [13], 3,5,6-trihydroxysitostane (**6**) [14], stigmastane-3β,5α,6β-triol-3,6-diacetate (**7**) [15] were identified by comparison of their spectroscopic data with the literature values.

The anti-inflammatory activity of all isolates were evaluated. The results (Table 2) showed that compounds **1**–**3** exhibited potent inhibitory activity against NO and TNF-α production in RAW264.7 cells and the steroids were inactive with the IC_50_ values more than 20 µM.

**Table 2:** IC_50_ values of the active compounds **1**–**3** against NO and TNF-α production in RAW264.7 cells.

Compound	IC_50_ (μM)	
	NO	TNF-α
**1**	19.2	6.2
**2**	6.7	5.6
**3**	13.8	8.9
Celastrol ^a^	1.1	0.9

^a^ Positive control for NO and TNF-α production.

## 3. Experimental

### 3.1. General

Optical rotations were measured on a Rudolph Autopol I automatic polarimeter. IR spectra were determined on a Bruker Tensor 37 infrared spectrophotometer. NMR spectra were measured on a Bruker AM-400 spectrometer at 25 °C. ESIMS was measured on a Finnigan LC QDECA instrument, and HRESIMS was performed on a Waters-Micromass Q-TOF. A Shimadzu LC-20 AT equipped with a SPD-M20A PDA detector was used for HPLC. A YMC-pack ODS-A column (250 × 10 mm, S-5 µM, 12 nm) was used for semipreparative HPLC separation. Silica gel (300–400 mesh, Qingdao Haiyang Chemical Co., Ltd., Qingdao city, China), C_18_ reversed-phase silica gel (12 nm, S-50 µM, YMC Co., Ltd., Taiwan Chu-Pei city, China), Sephadex LH-20 gel (Amersham Biosciences, Piscataway, NJ, USA) and MCI gel (CHP20P, 75–150 µM, Mitsubishi Chemical Industries Ltd., Tokyo, Japan.) were used for column chromatography. All solvents used were of analytical grade (Guangzhou Chemical Reagents Company, Ltd., Guangzhou, China). All cell lines were obtained from the China Center for Type Culture Collection of the Chinese Academy of Sciences.

### 3.2. Plant Material

Plant of *F. fimbriata* were collected in July 2010 from Yunnan Province, China, and were identified by one of the authors (Prof. D. P. Yang). A voucher specimen (accession number: LSJSH201010) has been deposited at the School of Pharmaceutical Sciences, Sun Yat-sen University.

### 3.3. Extraction and Isolation

The air-dried powder of the leaves of *F. fimbriata* (1 kg) was extracted with 95% EtOH (3 × 10 L) at room temperature (rt) to give 80 g of crude extract. The extract was suspended in H_2_O (1 L) and successively partitioned with petroleum ether (PE, 3 × 1 L), EtOAc (3 × 1 L) and n-BuOH (3 × 1 L), respectively. The PE extract (15 g) was subjected to silica gel column chromatography (CC) and eluted with PE/dichloromethane (0:10→10:0) successively to afford three fractions (I–III). Fraction I (2.5 g) was chromatographed over an silica gel column eluting with PE/CH_2_Cl_2_ (0:10→10:0) to afford five fractions (Fr. Ia–Ie), Fr. Ia was then subjected to silica gel column eluting with PE/CH_2_Cl_2_ (0:10→10:0) and obtained compound **4** (113 mg). Fraction III (3.5 g) was chromatographed over an silica gel column eluting with PE/Acetone (0:10→10:0) to afford five fractions (Fr. IIIa–IIIe). Fr. IIIa (1.0 g) was separated by silica gel CC (PE/CH_2_Cl_2_, 50:1→10:1) to afford five fractions (Fr. IIIaa–IIIae). Fr. IIIac was subjected to Rp-C_18_ CC using a gradient of MeOH/H_2_O (v/v from 7:3 to 10:0) to yield 6 (22 mg) and another fraction, which after chromatography on a Sephadex LH-20 column using CHCl_3_/MeOH (1:1) as eluent to obtain 7 (23 mg). The EtOAc extract (42 g) was subjected to MCI gel CC eluted with a MeOH/H_2_O gradient (3:7→10:0) to afford three fractions (XI–XIII). Fraction XI (6.5 g) was subjected to silica gel CC (PE/EtOAc, 2:1→0:1) to give three fractions (XIa–XIc). Fr. XIa (2.1 g) was separated by silica gel CC (PE/EtOAc, 2:1), followed by semi-preparative HPLC (CH_3_OH/H_2_O, 8:2, 3 mL/min) to give **1** (6 mg). Fr. XIc (1.3 g) was separated by Rp-C_18_ silica gel CC (MeOH/H_2_O, 5:5→0:0) to yield **2** (8 mg). Fraction XII (5.5 g) was subjected to silica gel CC (CHCl_3_/MeOH, 30:1→0:1) to give three fractions (XIIa–XIIc). Fr. XIIb (2.5 g) was subjected to silica gel CC (PE/CHCl_3_, 1:1→0:1) to give three fractions (XIIb_1_–XIIb_4_). Fr. XIIb_1_ (0.9 g) was subjected to Rp-C_18_CC (MeOH/H_2_O, 2:8→10:0) and followed by silica gel CC (EtOAc/acetone, 3:1→0:1) to afford **5** (38 mg). Fr. XIIb_4_ (1.2 g) was subjected to Rp-C_18_ CC (MeOH/H_2_O, 0:10→10:0) and Sephadex LH-20 (EtOH) to yield **3** (25 mg).

### 3.4. Spectral Data

*Flickinflimilin A* (**1**). Colorless oil; 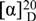 −46.2° (*c* 0.24, CHCl_3_); UV (MeOH) λ_max_ (log ε) 242 (2.18) nm, 203 (2.46) nm; CD (*c* 3.4 × 10^−3^ M, CH_3_CN), *λ*_max_ (∆ε) 242 (−0.26), 278 (−0.01), and 323 (−0.07); IR (KBr) ν_max_ 3437, 1718, 1591, 1461, 1378, 1129, 1038, 949, and 764 cm^−1^; ^1^H and ^13^C-NMR data, see Table 1; positive ESIMS *m*/*z* 315.3 [M + Na]^+^, 607.6 [2M + Na]^+^; HREIMS *m*/*z* 315.1943 [M + Na]^+^ (calcd for C_1__8_H_8_O_3_Na, 315.1936).

*Flickinflimilin B* (**2**). White amorphous powder; 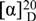 +16.0° (*c* 0.50, MeOH); UV (MeOH) λ_max_ (log ε) 207 (2.94), 251 (2.45) nm; CD (*c* 1.7 × 10^−3^ M, CH_3_CN), λ_max_ (∆ε) 207 (−6.64), 251 (+2.29), 270 (−0.34), 300 (+2.19), and 337 (−3.08); IR (KBr) ν_max_ 3440, 1708, 1600, 1500, 1476, 1129, 767, and 750 cm^−1^; ^1^H and ^13^C-NMR data, see Table 1; positive ESIMS *m*/*z* 289.1 [M + H]^+^; negative ESIMS*m*/*z* 333.2 [M + HCOO]^−^; HRESIMS *m*/*z* 311.1987 [M + Na]^+^ (calcd for C_1__8_H_24_O_3_Na, 311.1989).

*Flickinflimilin C* (**3**). Colorless oil; 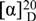 −55.0° (*c* 0.22, CHCl_3_); IR (KBr) ν_max_ 3445, 1714, 1646, 1461, 1379, 1257, 1126, 1085, and 950 cm^−1^; ^1^H and ^13^C-NMR data, see Table 1; positive ESIMS *m*/*z* 563.3 [M + Na]^+^, 1103.5 [2M + Na]+; negative ESIMS *m*/*z* 585.3 [M + HCOO]^−^, 1125.8 [2M + HCOO]−; HRESIMS *m/z* 585.2929 [M + HCOO]^−^ (calcd for C_2__9_H_4__5_O_1__2_, 585.2923).

### 3.5. Computational Methods for Electronic Circular Dichroism

Molecular mechanics calculations were carried out with Spartan’14 software package (Wavefunction Inc., Irvine, CA, USA, 2013) and quantum chemical computations were run with Gaussian 09 program package (Gaussian, Inc., Pittsburgh PA, USA, 2011) using default grids and convergence criteria. MMFF conformational search generated conformers within a 10 kcal/mol energy window were optimized using DFT method at B3LYP/6-31G (d) level. Frequency calculations were run at the same level to verify that each optimized conformer was a true minimum and to estimate their relative thermal free energies (∆*G*) at 298.15K. The TDDFT calculations were performed using the hybrid B3LYP functional, and Ahlrichs’ basis sets SVP (split valence plus polarization) and TZVP (triple zeta valence plus polarization). The number of excited states per each molecule was 20–30. Solvent effects were taken into account by using polarizable continuum model (PCM). CD spectra were generated by the program SpecDis (University of Würzburg, Würzburg, Germany, 2012) using a Gaussian band shape with 0.28 eV exponential half-width from dipole-length dipolar and rotational strengths; the difference with dipole-velocity values was negligible (<10%) for most transitions.

### 3.6. Determination of Sugar Configuration [16]

Compound **3** (6 mg) was refluxed with 2 M HCl (2 mL, dioxane/H_2_O, 1:1) at 100 °C for 4 h. After removing the dioxane under vacuum, the solution was then diluted with H_2_O and then extracted with EtOAc (3 × 1 mL). The EtOAc layer was evaporated under vacuum, then subjected to CC over silica gel eluteing with CHCl_3_:MeOH (30:1) to afford **3a**. The aqueous layer was evaporated under vacuum, diluted repeatedly with H_2_O, evaporated under vacuum to obtain neutral residue, and then analyzed by TLC over silica gel (Me_2_CO/*n*-BuOH/H_2_O, 6:3:1) together with authentic sugar sample (glucose, R_f_ = 0.49). The remaining residue was dissolved in pyridine (200 µL), to which 2 mg of l-cysteine methyl ester hydrochloride was added. The mixture was stirred at 60 °C for 1 h; then 50 µL of *o*-tolyl isothiocyanate was added, and the mixture was stirred at 60 °C for another 1 h. The reaction mixture was directly analyzed by standard C_18_ HPLC [a YMC-pack ODS-A column (250 × 10 mm, S-5 µM, 12 nm), CH_3_CN/H_2_O, 25:75, 3 mL/min]. The peak (*t*_R_ = 19.0 min) coincided with a derivative of d-glucose, as compared with authentic d-glucose with *t*_R_ at 19.1 min.

### 3.7. Chemical Transformation of **3a** to **3c** [17]

To a stirred solution of **3a** (10 mg) in MeOH (2 mL), NaOH (1 mg) was added. The mixture was stirred at room temperature for 0.5 h to obtain **3b**, and then **3b** (7.2 mg) was transferred into a clean NMR tube and was dried completely under the vacuum of an oil pump. Deuterated pyridine (0.55 mL) and 4-methoxybenzoyl chloride (12 µL) were added into the NMR tube immediately under dry conditions, and then the NMR tube was shaken carefully to mix the sample and 4-methoxybenzoyl chloride evenly. The reaction NMR tube was permitted to stand at 45 °C and monitored by ^1^H-NMR. The reaction was found to be completed after 2 h. ^1^H-NMR data of the 4-methoxybenzoate derivative (**3c**) of **3b** was obtained from the reaction NMR tube directly. The reaction mixtures were transferred from the NMR tube and subjected to Sephadex LH-20 CC eluting with CHCl_3_:MeOH (1:1). CD (CH_3_OH, ∆ε) 212 (−17.20), 243 (+2.85), 267 (−18.41), 297 (+1.12) nm; ^1^H-NMR (CDCl_3_, 400 MHz) δ_H_ 5.43, (1H, s, H-14), 5.40, [1H, (ddd, *J* = 2.1, 4.3, 12.4 Hz, H-2)], 4.15, [1H, (dd, *J* = 7.6, 14.2 Hz, H-16a)], 3.67, [1H, (d, *J* = 2.1 Hz, H-3)], 3.15, [1H, (dd, *J* = 7.6, 14.2 Hz, H-16b)], 2.29–2.42, (4H, m, H-6, H-7), 1.96–2.14, (4H, m, H-1, H-12), 1.83, (1H, m, H-9), 1.44, (1H, m, H-5), 1.07, (3H, s, H-17), 1.01, (3H, s, H-18), 0.92, (3H, s, H-19), 0.88, (3H, s, H-20), [7.97–8.05, (6H, m), 6.91–6.96, (6H, d, *J* = 8.5 Hz ), 3.86–3.87, (9H, s, CH_3_O-p), p-methoxybenzoyl]; ^13^C-NMR (CDCl_3_, 100 MHz) δ_C_ 207.2 (C-15), 142.1 (C-8), 124.1 (C-14), 76.8 (C-3), 71.3 (C-2), 66.7 (C-16), 50.8 (C-9), 47.7 (C-5), 47.3 (C-13), 39.5 (C-10), 38.7 (C-4), 36.2 (C-7), 35.6 (C-12), 32.6 (C-1), 28.6 (C-18), 27.2 (C-17), 22.6 (C-19), 21.6 (C-11), 20.2 (C-6), 15.4 (C-20), (165.7, 165.5, 163.6, 163.5, 132.0, 131.6, 113.7, 113.6, 55.4) (*p*-methoxybenzoyl); negative ESIMS *m*/*z* 737.3 [M − H]^−^.

### 3.8. Biological Assays

#### 3.8.1. Cytotoxic Assay [18]

The RAW264.7 cell line was obtained from ATCC (Manassas, VA, USA), and was cultured in DMEM medium (Hyclone, Logan, UT, USA), supplemented with 10% fetal bovine serum (FBS, Hyclone) at 37 °C in a humidified atmosphere with 5% CO_2_. Cell viability was assessed by MTT (Sigma, St. Louis, MO, USA). Briefly, 100 µL of adherent cells with an initial density of 1 × 10^5^ cells/mL were seeded into a 96-well plate and allowed to adhere for 24 h. Cells were exposed to the test compounds at various concentrations for 48 h. After the incubation, MTT (5 mg/mL) was added to each well, and the incubation continued for 4 h at 37 °C. The cells were lysed with 100 µL of 20% SDS–50% DMF after removal of the medium. The optical density of the lysate was measured at 595 nm in a 96-well microtiter plate reader (Bio-Rad 680, Hercules, CA, USA). The IC_50_ value of each compound was calculated by Reed and Muench’s method [19].

#### 3.8.2. Nitric Oxide Inhibitory Assay [20]

Inhibition of NO production was determined in a LPS-stimulated RAW264.7 macrophage cell line. Cells were seeded in 96-well plates (1 × 10^5^ cells/well) and allowed to adhere for 24 h at 37 °C in a humidified atmosphere containing 5% CO_2_. The medium was then replaced with fresh medium containing LPS (2 µg/mL) and test compounds at 10 µM, and the cells were incubated for 24 h. NO production was determined by measuring the accumulation of nitrite in the culture supernatant with Griess reagent (0.5% sulfanilamide and 0.05% naphthylene-diamide dihydrochloride in 2.5% H_3_PO_4_) and then allowed to stand for 5 min at rt. The absorbance at 540 nm was measured using a HTS 7000 microplate reader. The nitrite concentration in the medium was determined from the calibration curve (*r* = 0.9998) obtained by using different concentrations of sodium nitrite (NaNO_2_) in the culture medium as the standard. Blank correction was performed by subtracting the absorbance due to medium only from the absorbance reading of each well.

#### 3.8.3. Assay for the Production of Pro-inflammatory Cytokines (TNF-α) [21]

Compounds were dissolved in DMSO, and suspensions of RAW264.7 cells were cultured in complete RPMI 1640 medium (Hyclone) containing 10% FBS. The cultured cells were incubated with the tested compounds (10 µM) for 24 h, followed by LPS stimulation (2 µg/mL). Supernatants were collected to analyze cytokine levels. The mouse TNF-α ELISA Kit was used to determine the cytokine concentration in the culture supernatants.

## 4. Conclusions

Three new diterpenoids including two 15,16-dinor-*ent*-pimaranes and one diterpenoid glycoside, and four steroids were isolated from the leaves of *F. flimbriata*. The absolute configurations of the new compounds were determined by calculated ECD method, chemical transformation and exiton chirality method. All the isolates were screened for the anti-inflammation, the new compounds exhibited potent inhibitory against NO and TNF-α production in RAW264.7 cells.

## Supplementary Materials

Supplementary material can be accessed at http://www.mdpi.com/1420-3049/19/5/5863/s1.
