# Supplementary material for: Norditerpenoids from Flickingeria fimbriata and Their Inhibitory Activities on Nitric Oxide and Tumor Necrosis Factor-α Production in Mouse Macrophages
Source: Molecules. 2014 May 6;19(5):5863–75. doi: 10.3390/molecules19055863 (PMC6271523; doi:10.3390/molecules19055863)
Supplement: Supplementary file 1 [file molecules-19-05863-s001.pdf]

Supplementary Materials

Contents:

Figure S1. ^{1}H-NMR Spectrum of **1** in $CDCl_3$.
Figure S2. ^{13}C-NMR Spectrum of **1** in $CDCl_3$.
Figure S3. ^{1}H-^{1}H COSY Spectrum of **1** in $CDCl_3$.
Figure S4. HSQC Spectrum of **1** in $CDCl_3$.
Figure S5. HMBC Spectrum of **1** in $CDCl_3$.
Figure S6. NOESY Spectrum of **1** in $CDCl_3$.
Figure S7. ^{1}H-NMR Spectrum of **2** in Pyridine.
Figure S8. ^{13}C-NMR Spectrum of **2** in Pyridine.
Figure S9. ^{1}H-^{1}H COSY Spectrum of **2** in Pyridine.
Figure S10. HSQC Spectrum of **2** in Pyridine.
Figure S11. HMBC Spectrum of **2** in Pyridine.
Figure S12. NOESY Spectrum of **2** in Pyridine.
Figure S13. ^{1}H-NMR Spectrum of **3** in $CDCl_3$.
Figure S14. ^{13}C-NMR Spectrum of **3** in $CDCl_3$.
Figure S15. ^{1}H-^{1}H COSY Spectrum of **3** in $CDCl_3$.
Figure S16. HSQC Spectrum of **3** in $CDCl_3$.
Figure S17. HMBC Spectrum of **3** in $CDCl_3$.
Figure S18. NOESY Spectrum of **3** in $CDCl_3$.
Figure S19. ^{1}H-NMR Spectrum of **4** in $CDCl_3$.
Figure S20. ^{13}C-NMR Spectrum of **4** in $CDCl_3$.
Figure S21. ^{1}H-NMR Spectrum of **5** in $CDCl_3$.
Figure S22. ^{13}C-NMR Spectrum of **5** in $CDCl_3$.
Figure S23. ^{1}H-NMR Spectrum of **6** in $CDCl_3$.
Figure S24. ^{13}C-NMR Spectrum of **6** in $CDCl_3$.
Figure S25. ^{1}H-NMR Spectrum of **7** in $CDCl_3$.
Figure S26. ^{13}C-NMR Spectrum of **7** in $CDCl_3$.
Figure S27. The selected HMBC (H ⟶ C), ^{1}H-^{1}H COSY (H — H) and NOESY (H ↔ H) correlations.
Figure S28. The CD spectra of compounds **3** and **3c**.
Figure S29. The entire HMBC correlations and ^{1}H-^{1}H COSY (H — H) correlations of compound **1**.
Figure S30. The selected NOESY (H ↔ H) correlations of compound **3**.
Table S1. The purities of compounds **1**–**7**.

Figure S1. ^{1}H-NMR Spectrum of **1** in $CDCl_3$.

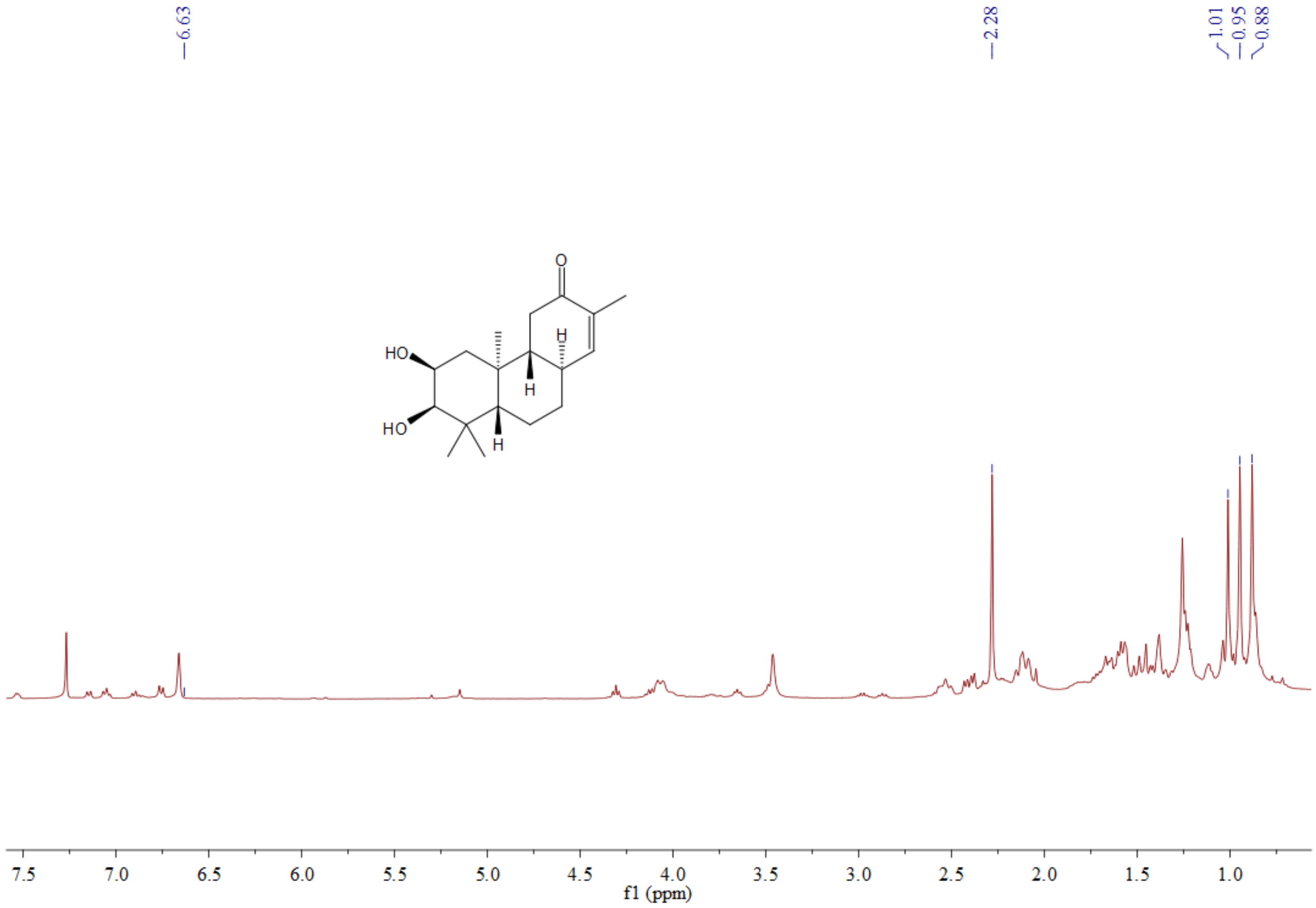

Figure S2. ^{13}C-NMR Spectrum of **1** in $CDCl_3$.

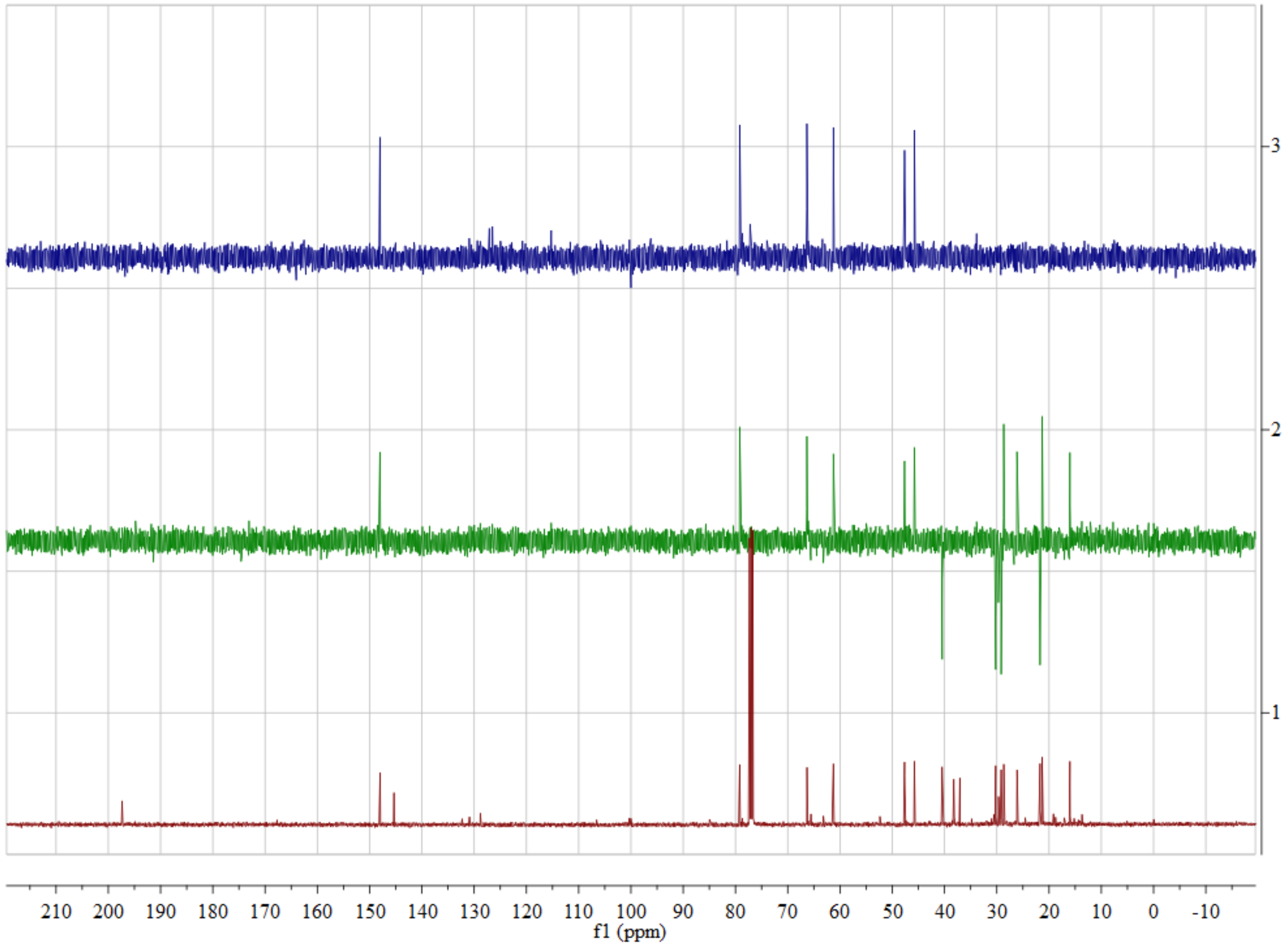

Figure S3. ^1H-^1H COSY Spectrum of **1** in $CDCl_3$.

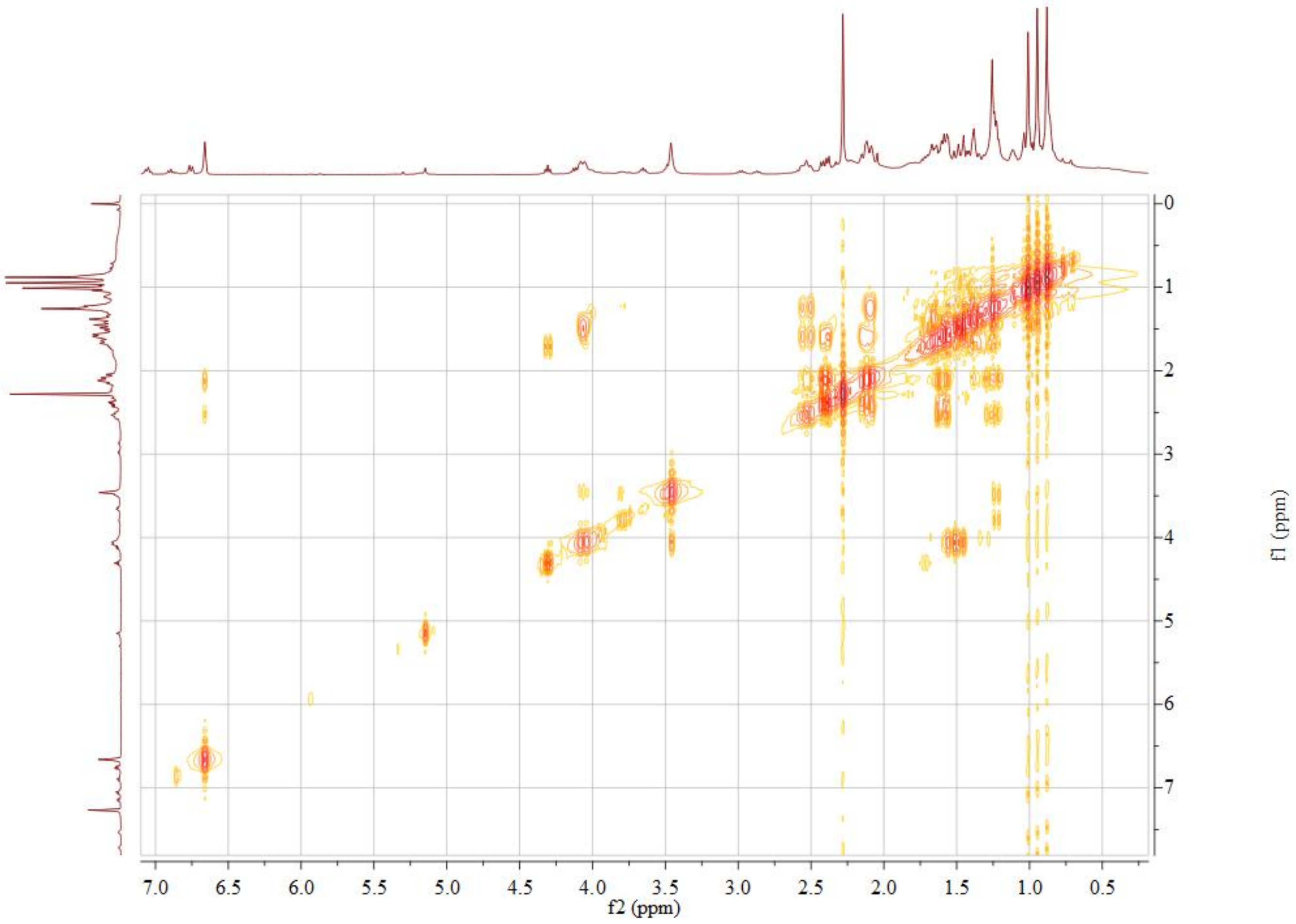

Figure S4. HSQC Spectrum of **1** in $CDCl_3$.

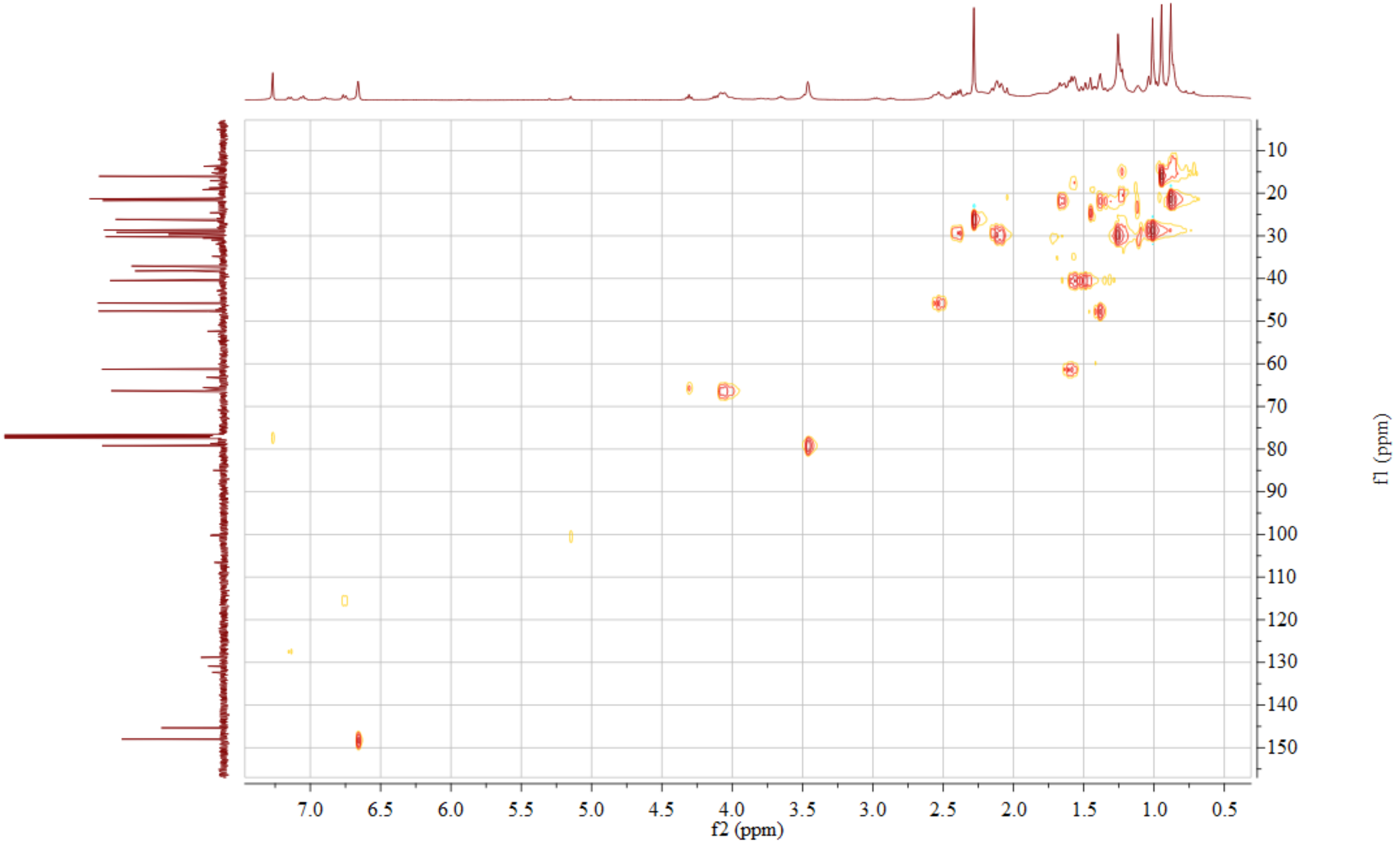

Figure S5. HMBC Spectrum of **1** in $CDCl_3$.

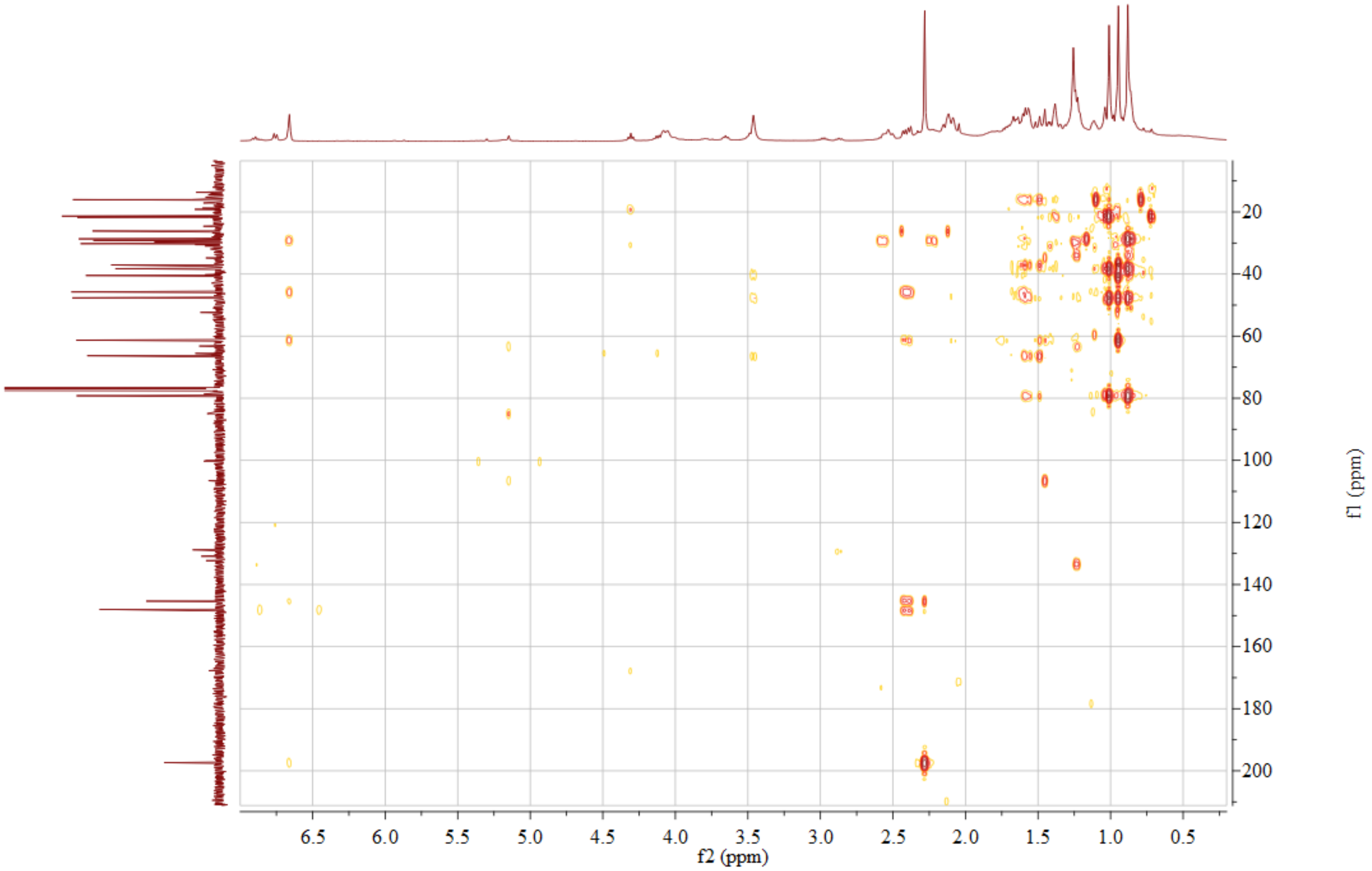

Figure S6. NOESY Spectrum of **1** in $CDCl_3$.

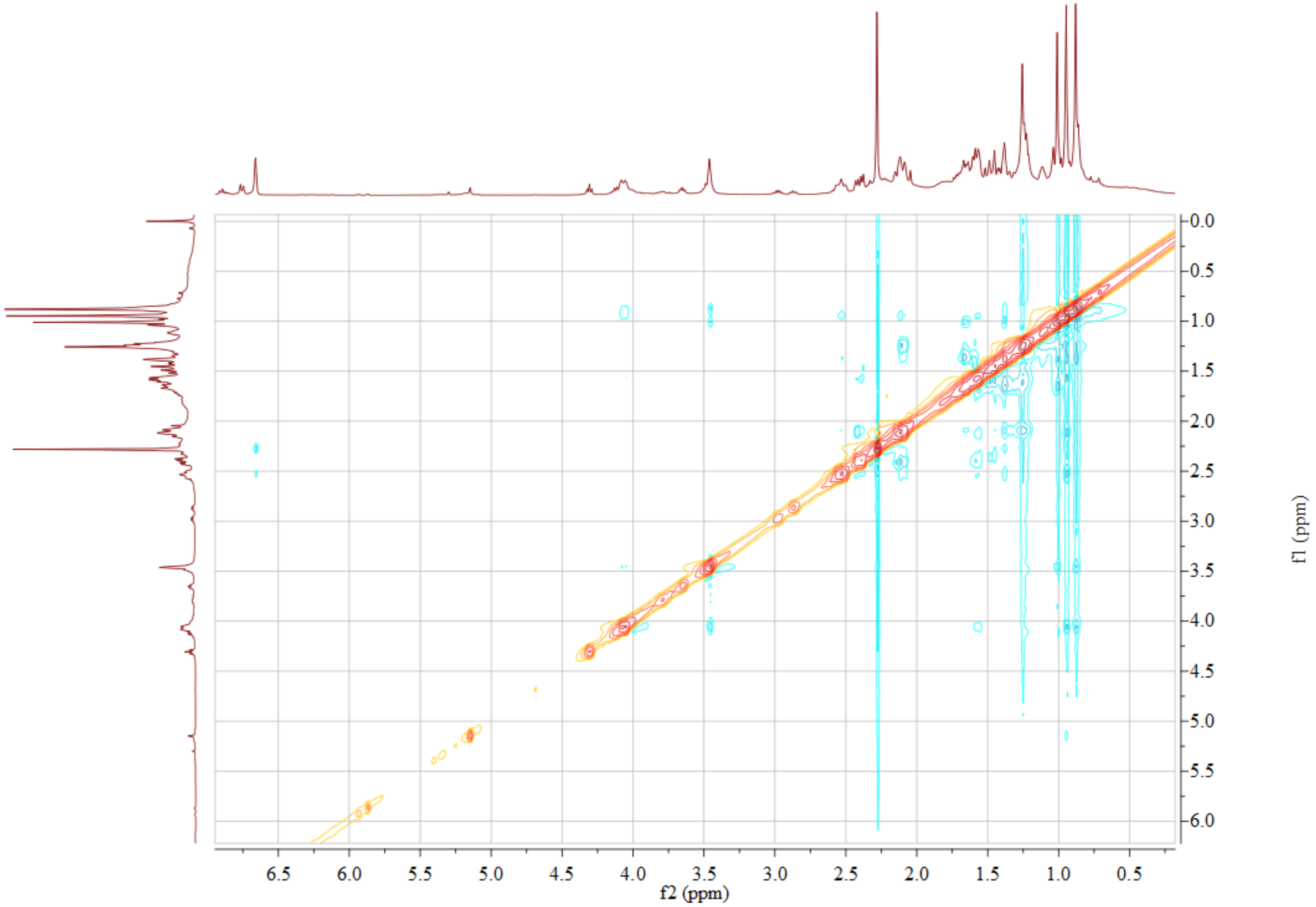

Figure S7. ^{1}H-NMR Spectrum of **2** in Pyridine.

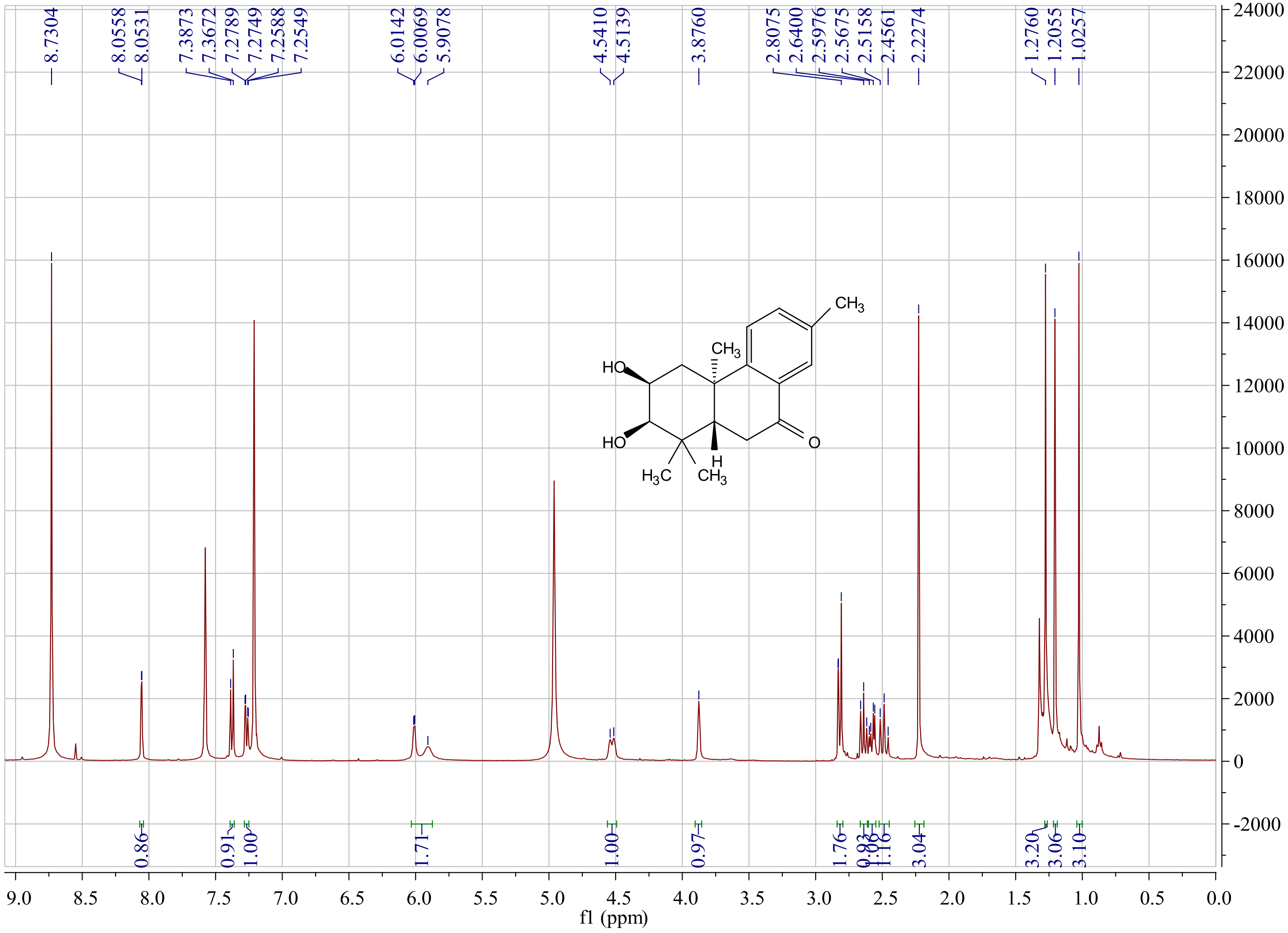

Figure S8. ^{13}C-NMR Spectrum of **2** in Pyridine.

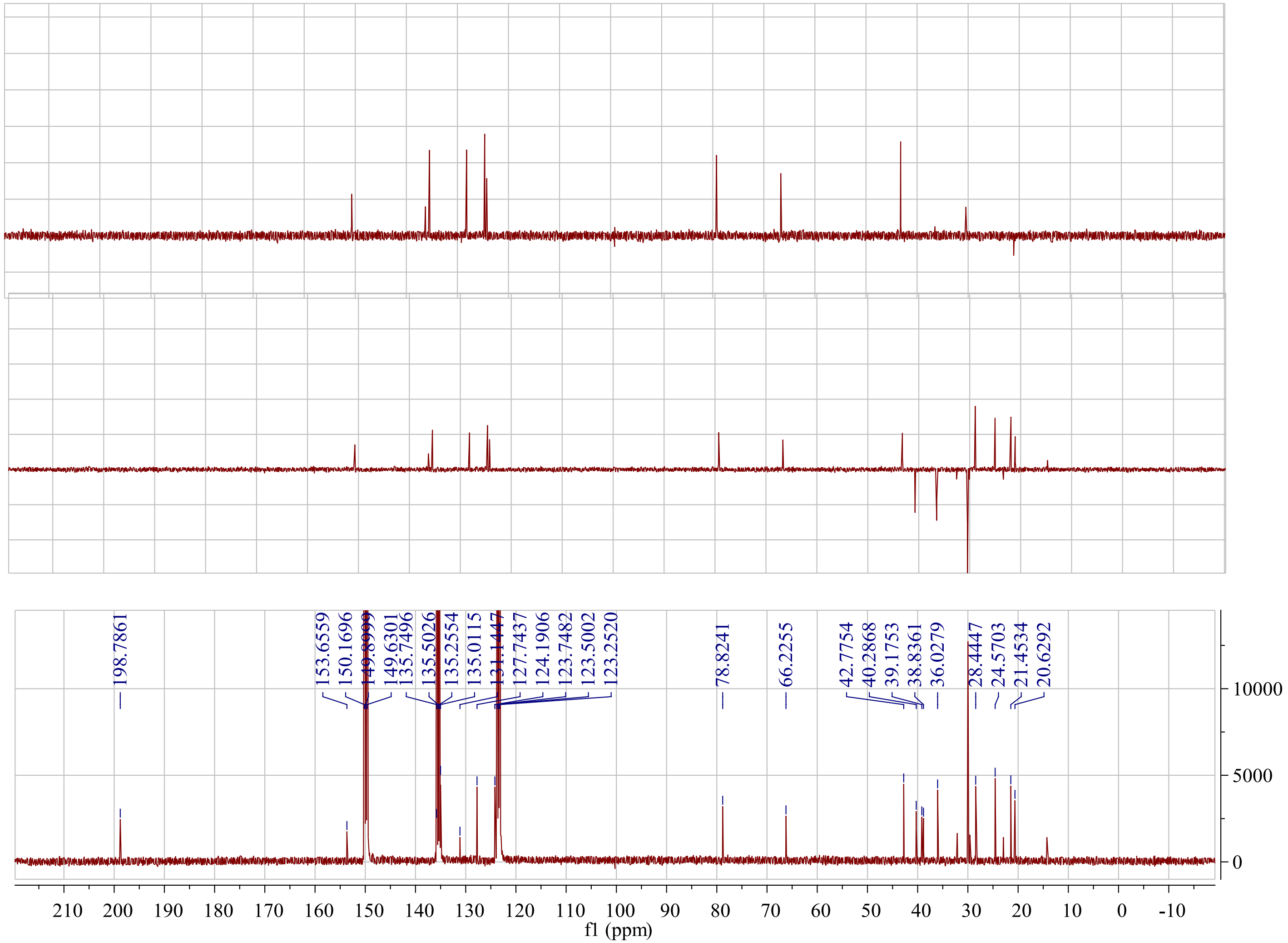

Figure S9. ^{1}H-^{1}H COSY Spectrum of **2** in Pyridine.

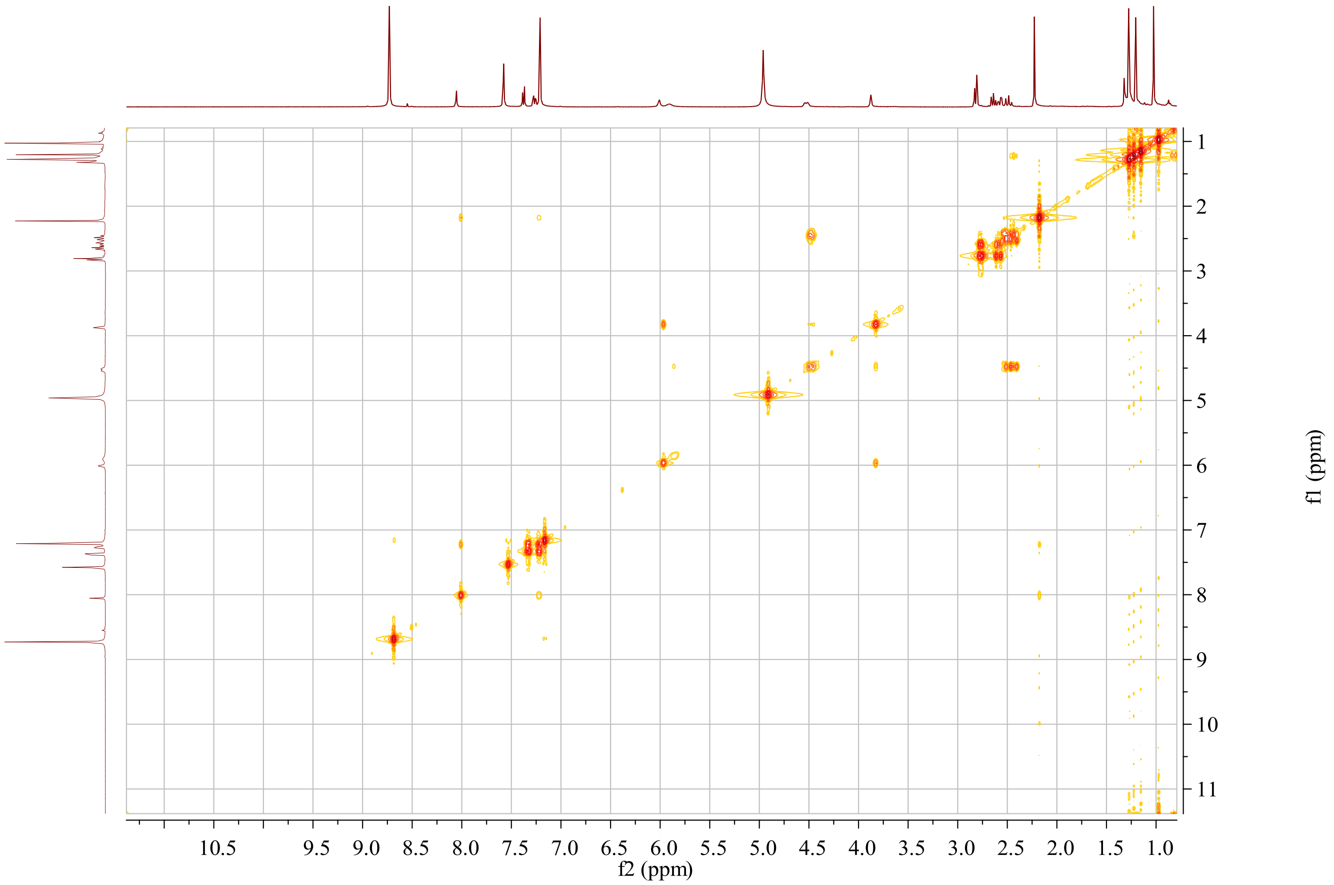

Figure S10. HSQC Spectrum of **2** in Pyridine.

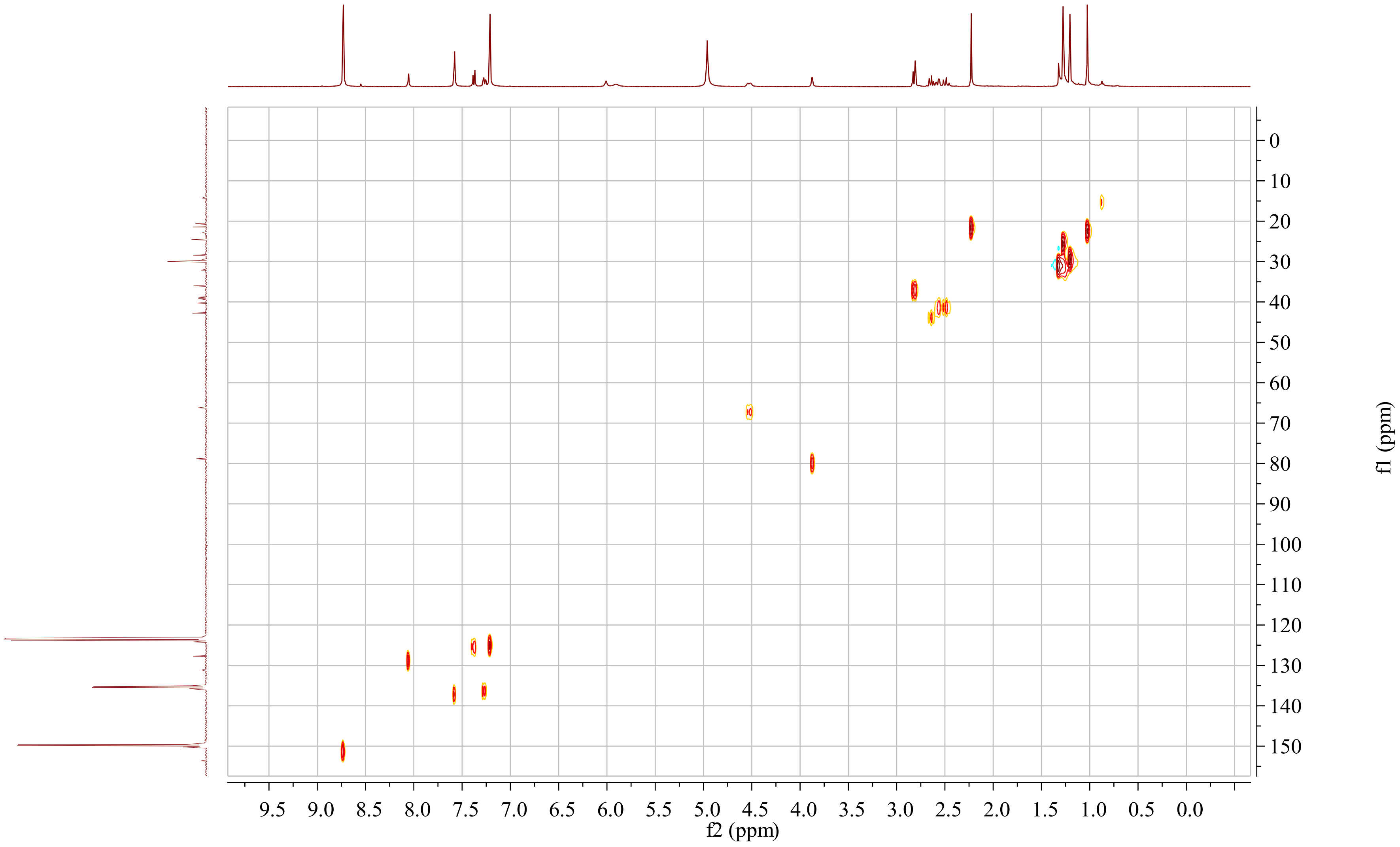

Figure S11. HMBC Spectrum of **2** in Pyridine.

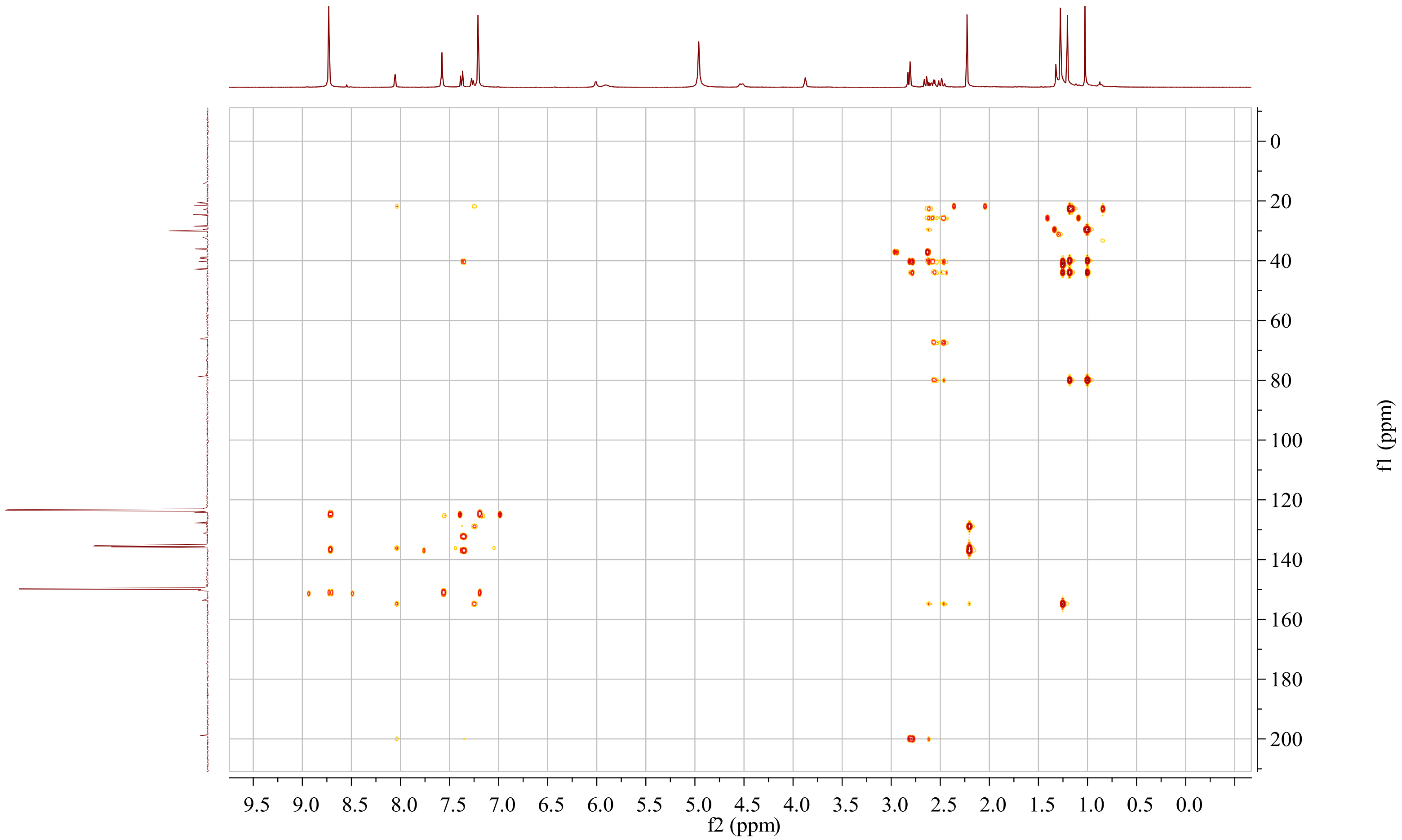

Figure S12. NOESY Spectrum of **2** in Pyridine.

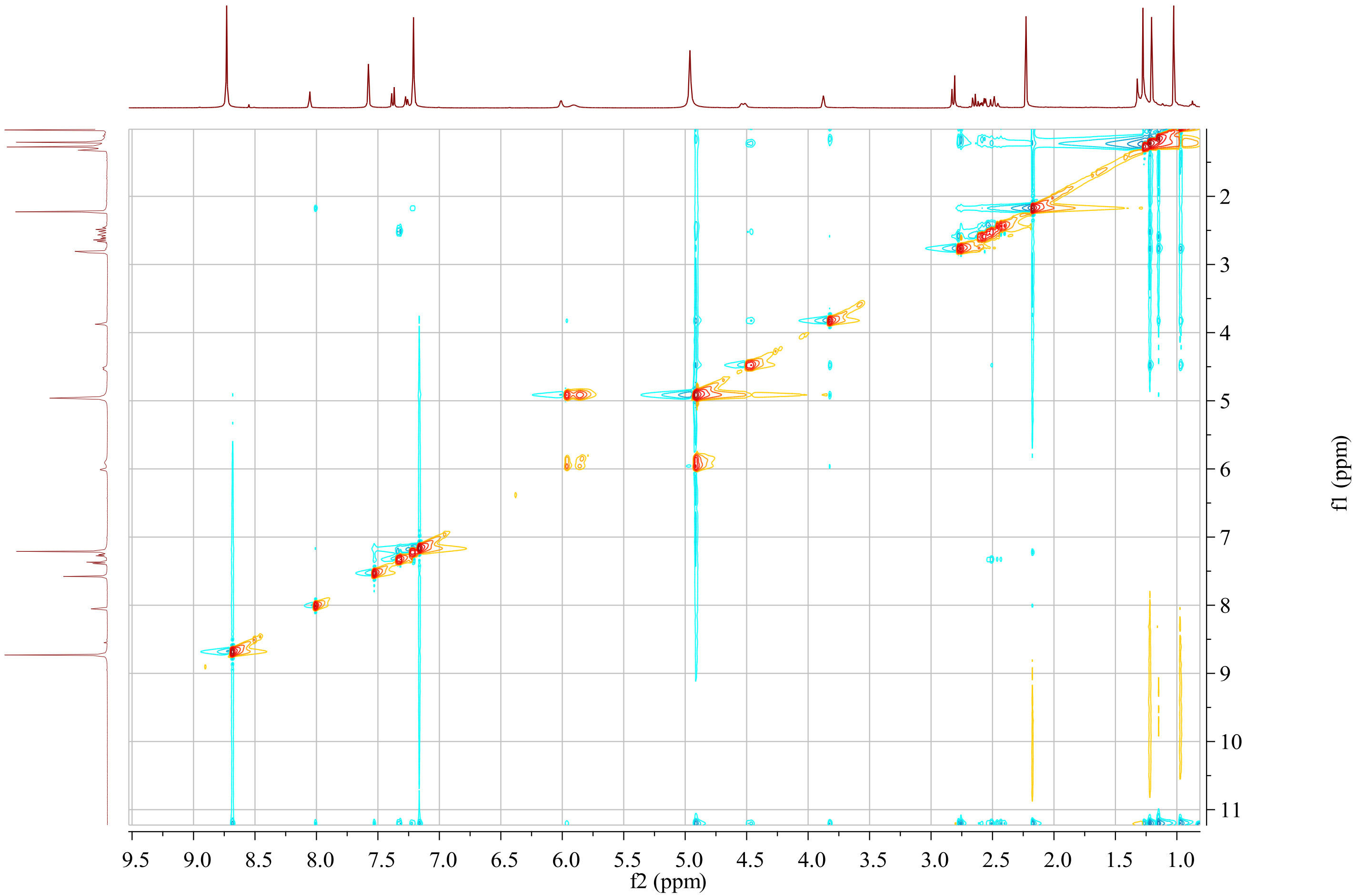

Figure S13. ^{1}H-NMR Spectrum of **3** in $CDCl_3$.

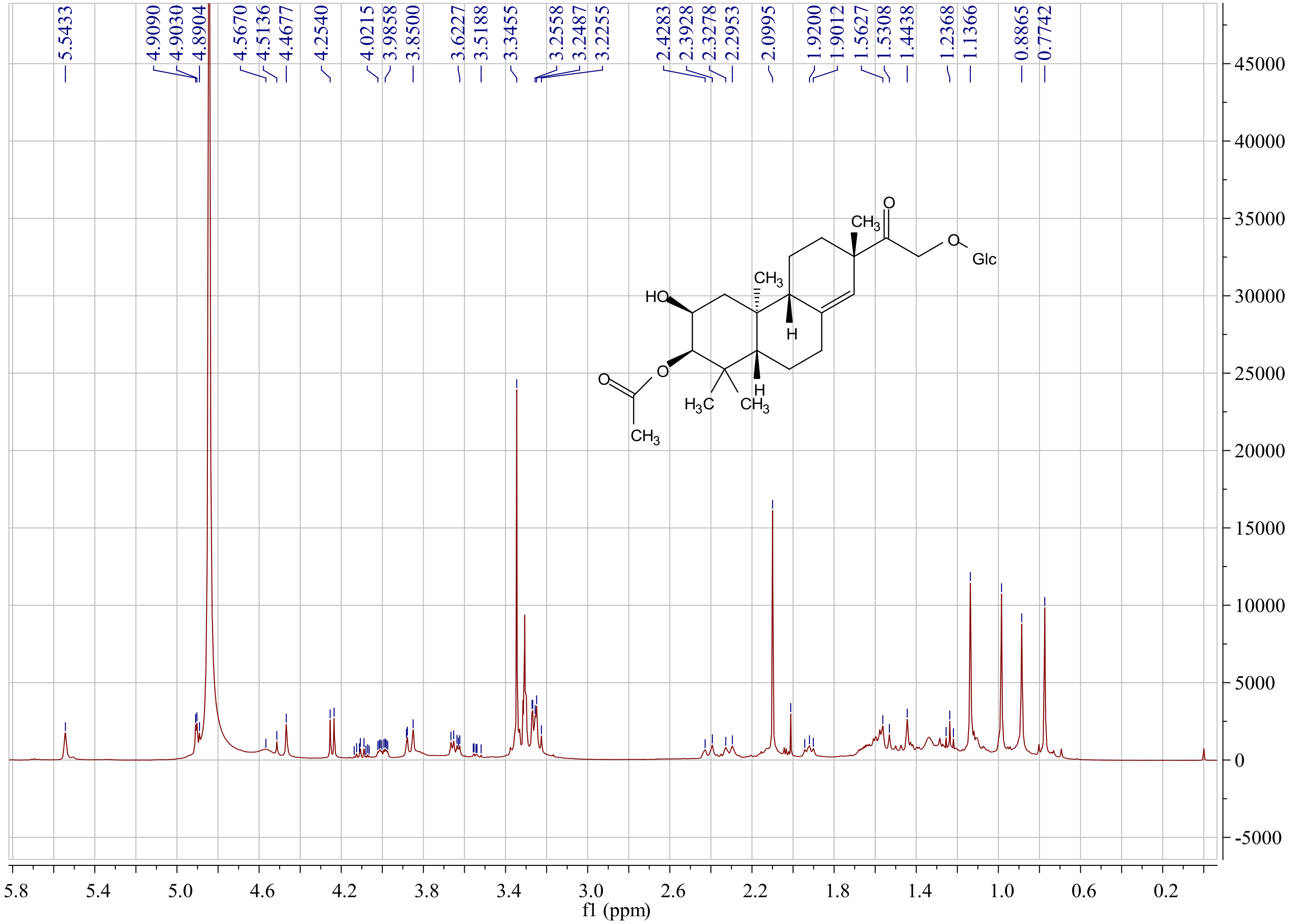

Figure S14. ^{13}C-NMR Spectrum of **3** in $CDCl_3$.

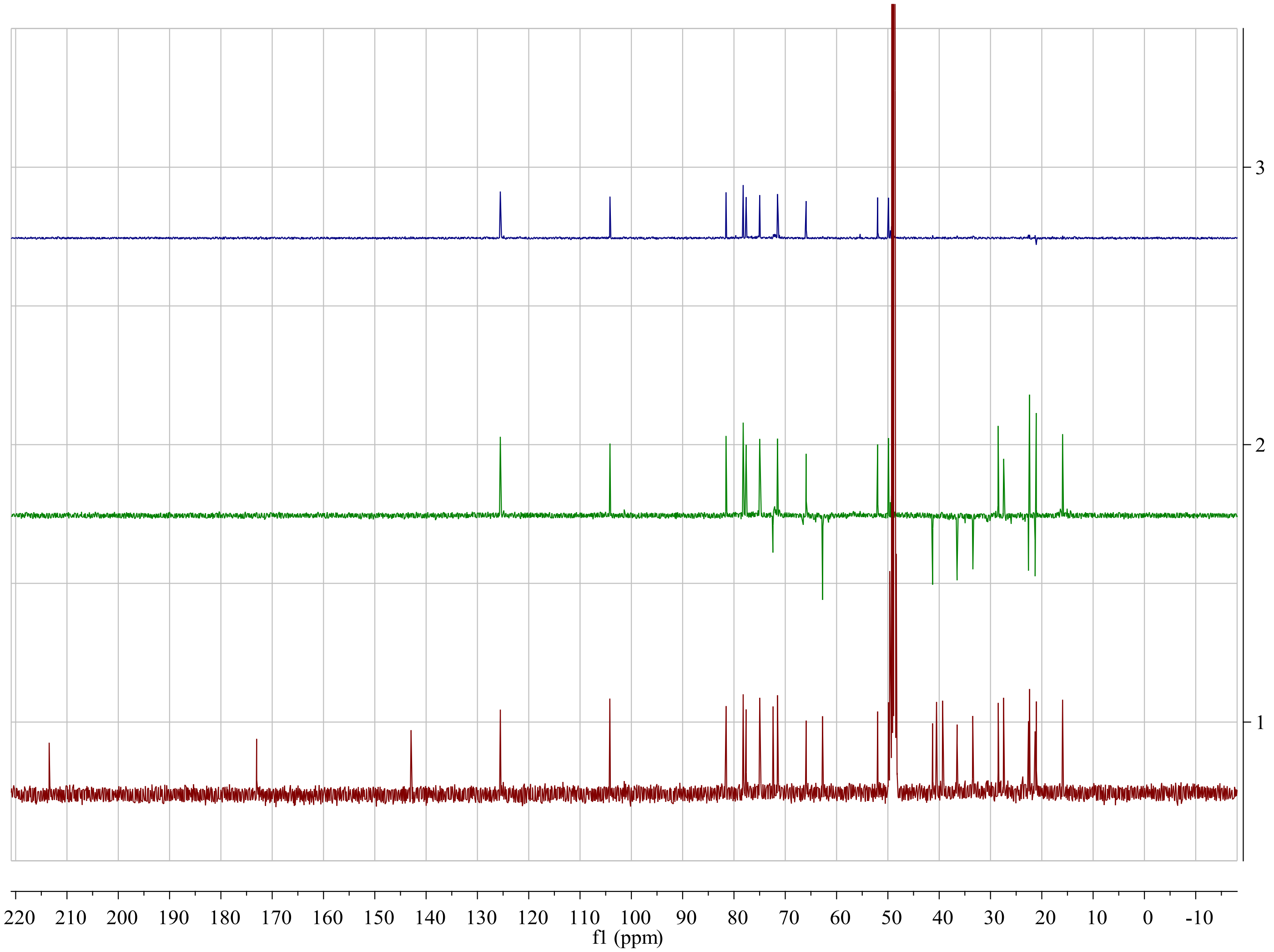

Figure S15. ^{1}H-^{1}H COSY Spectrum of **3** in $CDCl_3$.

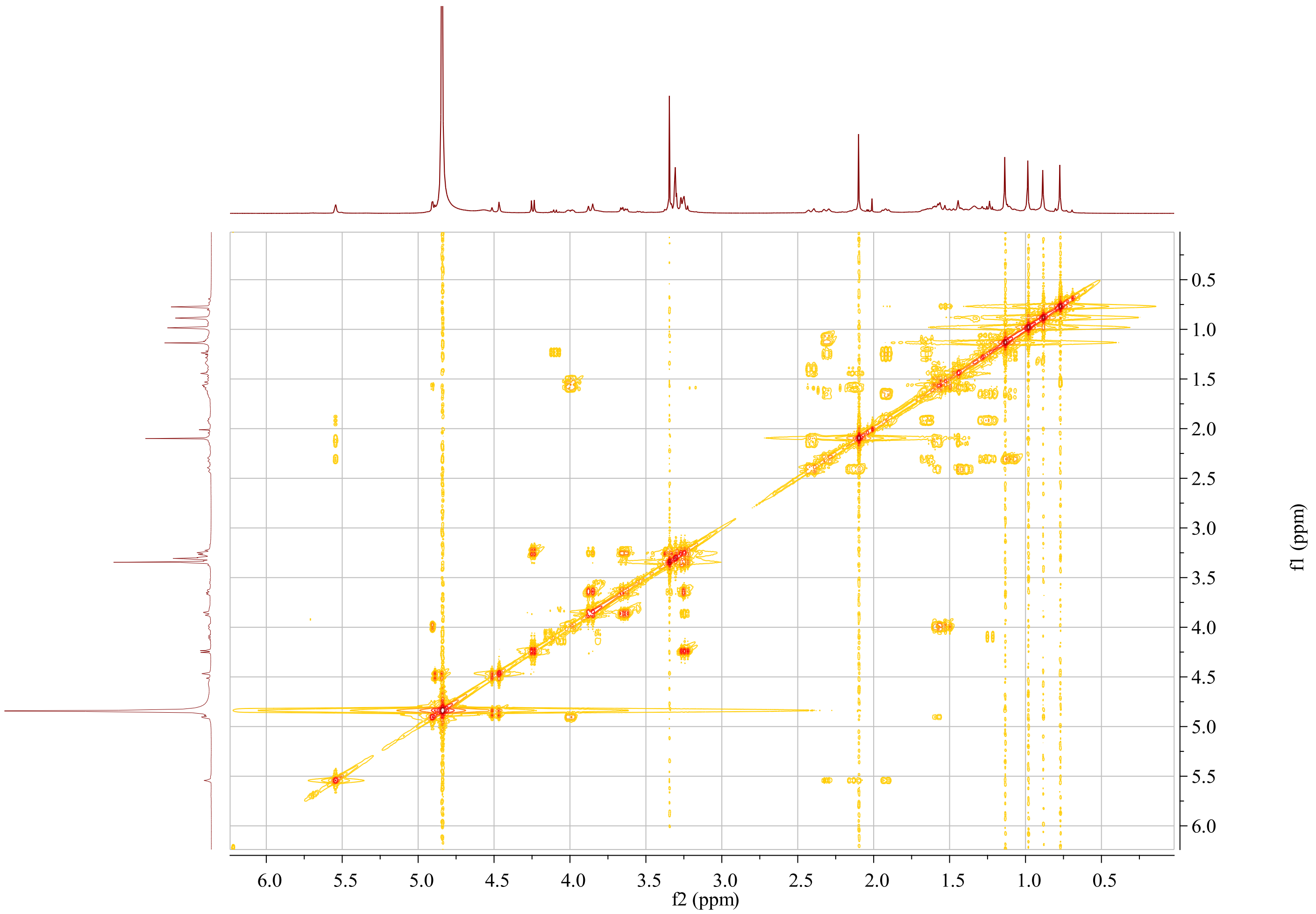

Figure S16. HSQC Spectrum of **3** in $CDCl_3$.

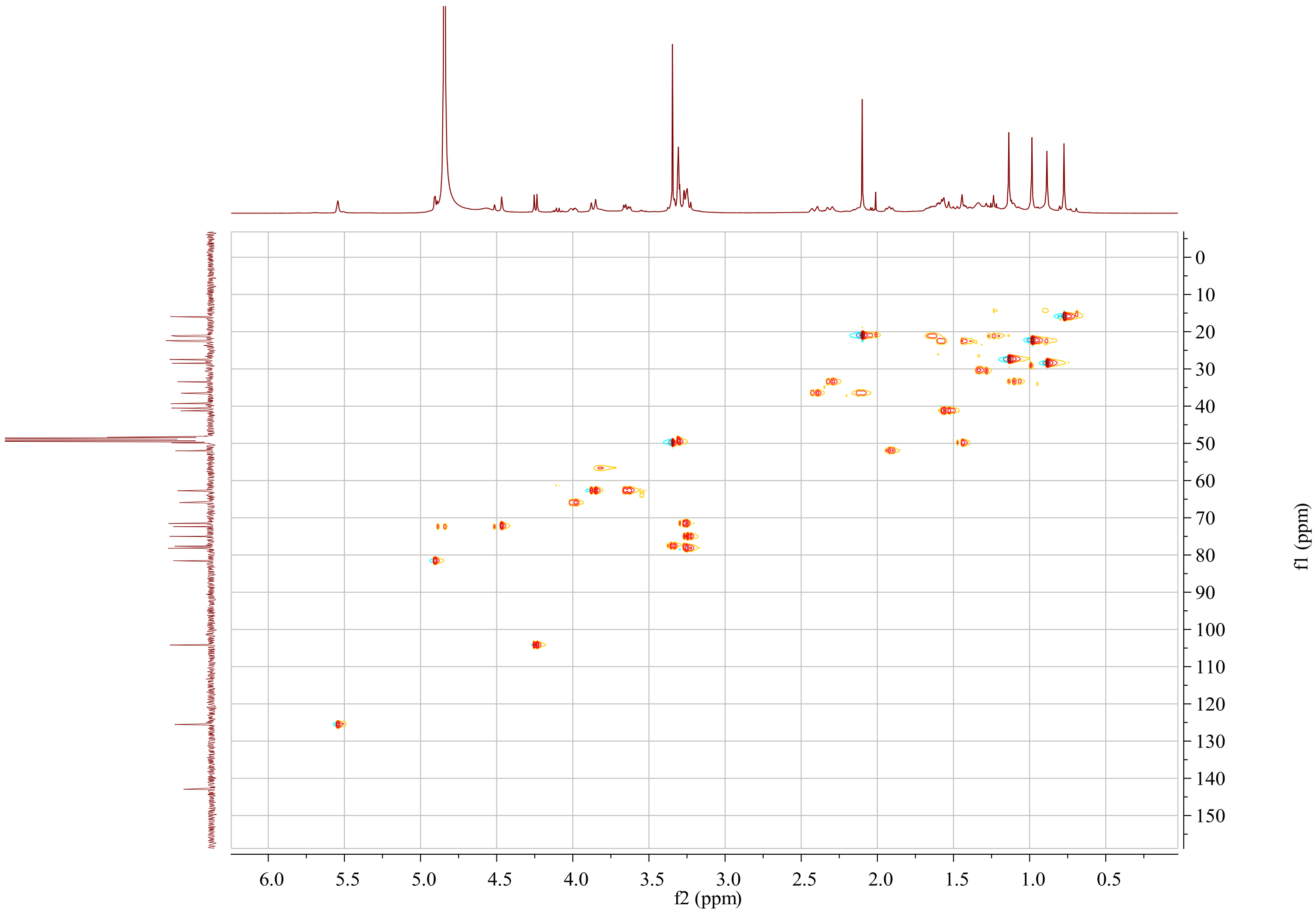

Figure S17. HMBC Spectrum of **3** in $CDCl_3$.

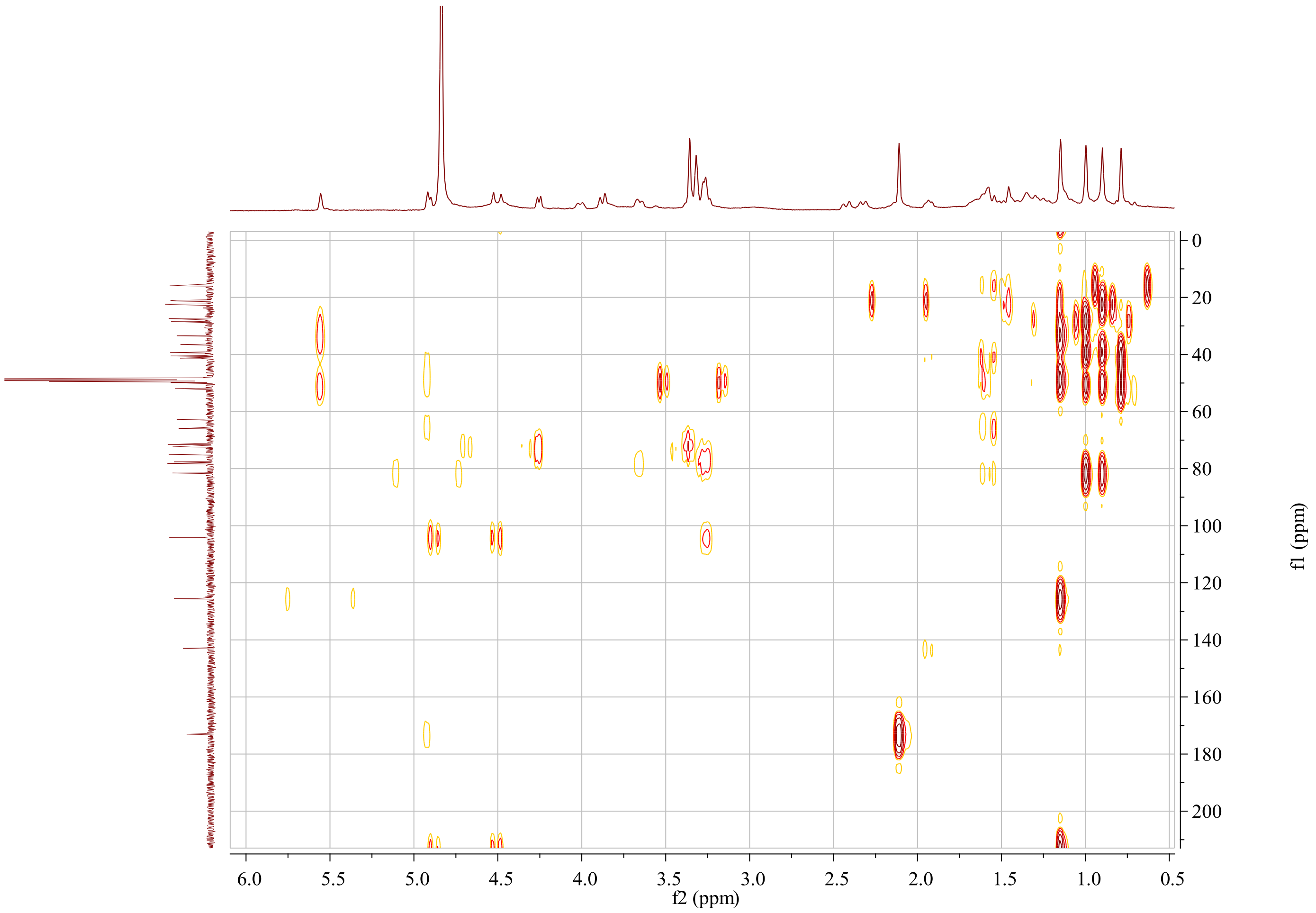

Figure S18. NOESY Spectrum of **3** in $CDCl_3$.

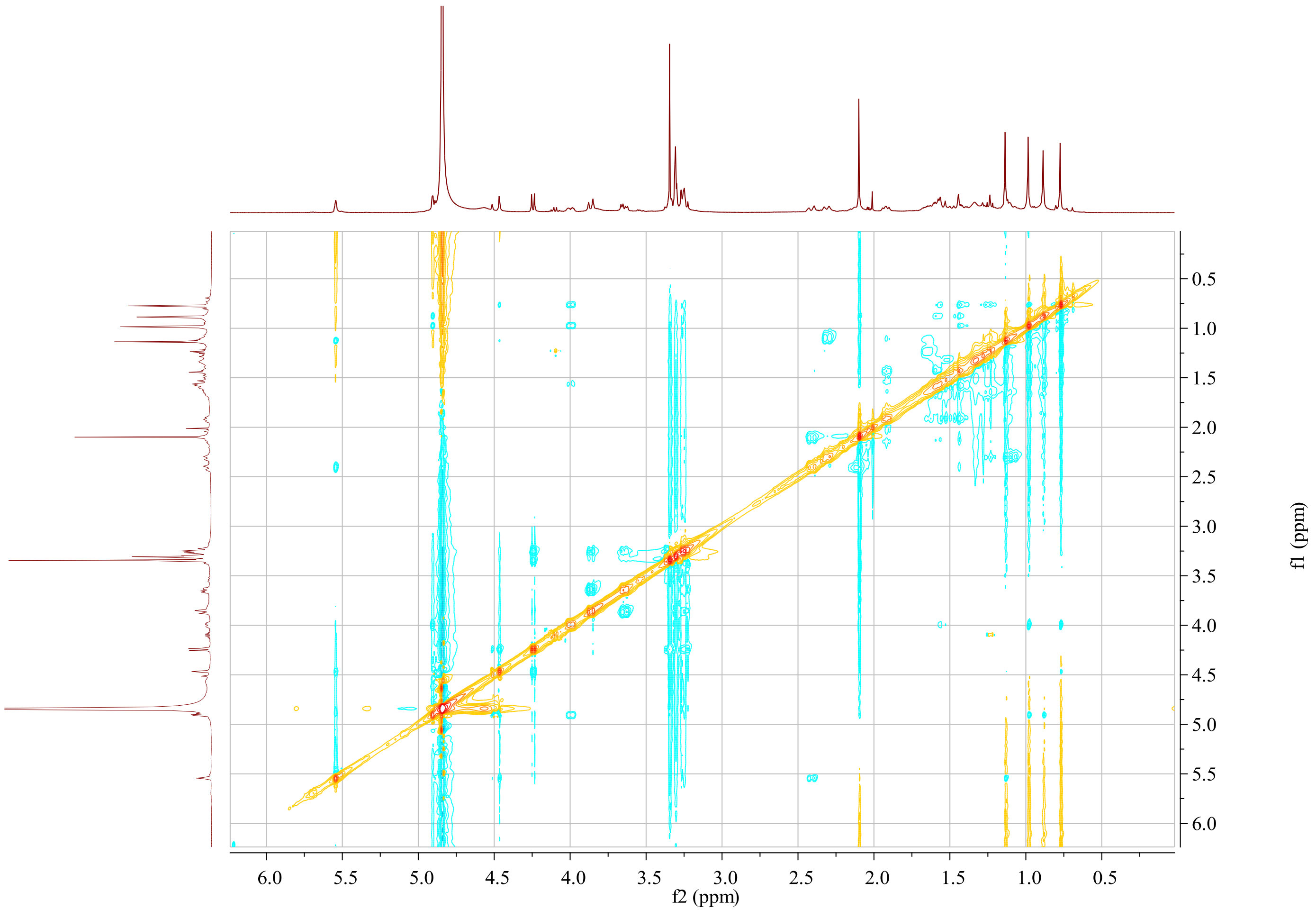

Figure S19. ^{1}H-NMR Spectrum of **4** in $CDCl_3$.

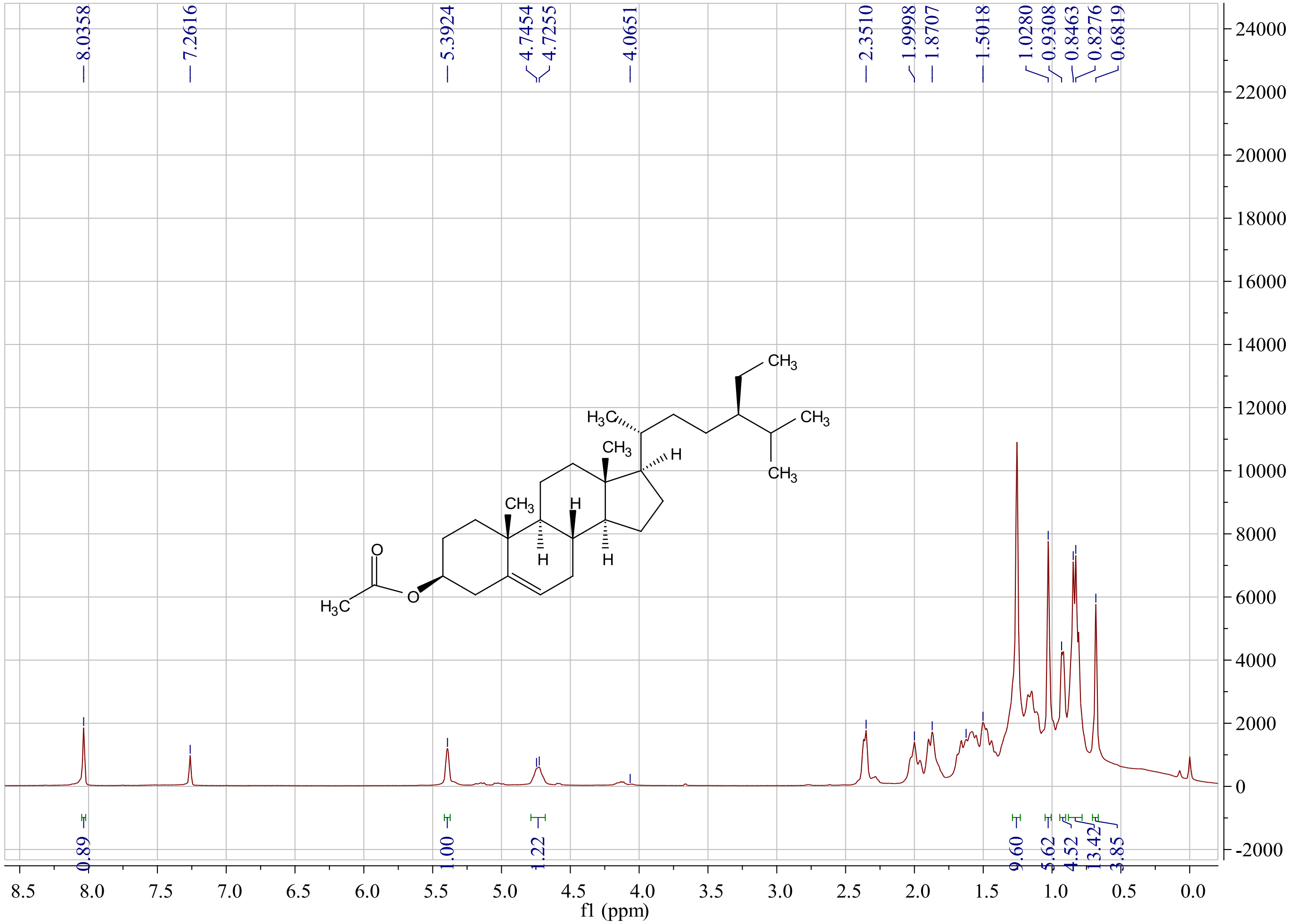

Figure S20. ^{13}C-NMR Spectrum of **4** in $CDCl_3$.

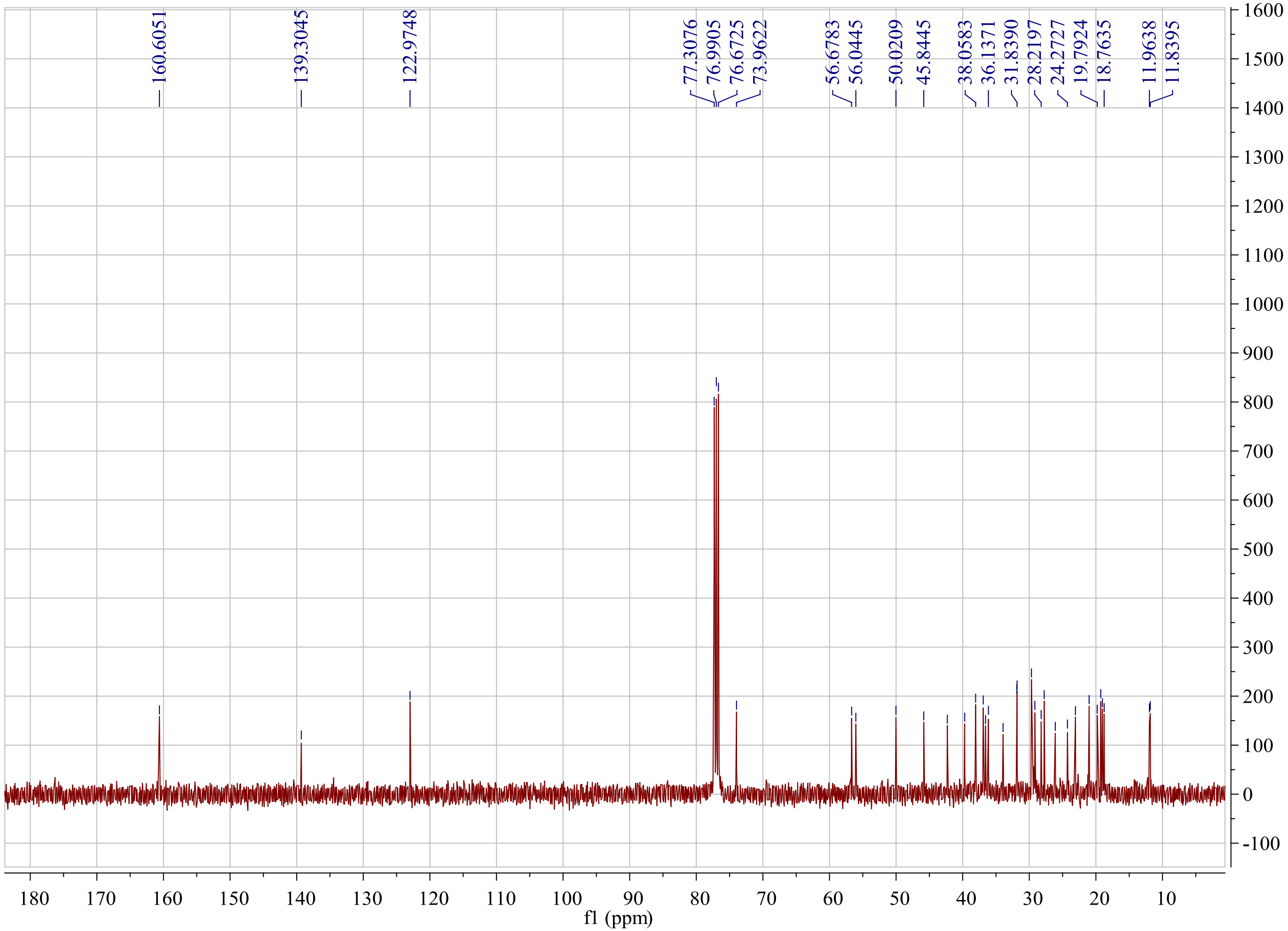

Figure S21. ^{1}H-NMR Spectrum of **5** in $CDCl_3$.

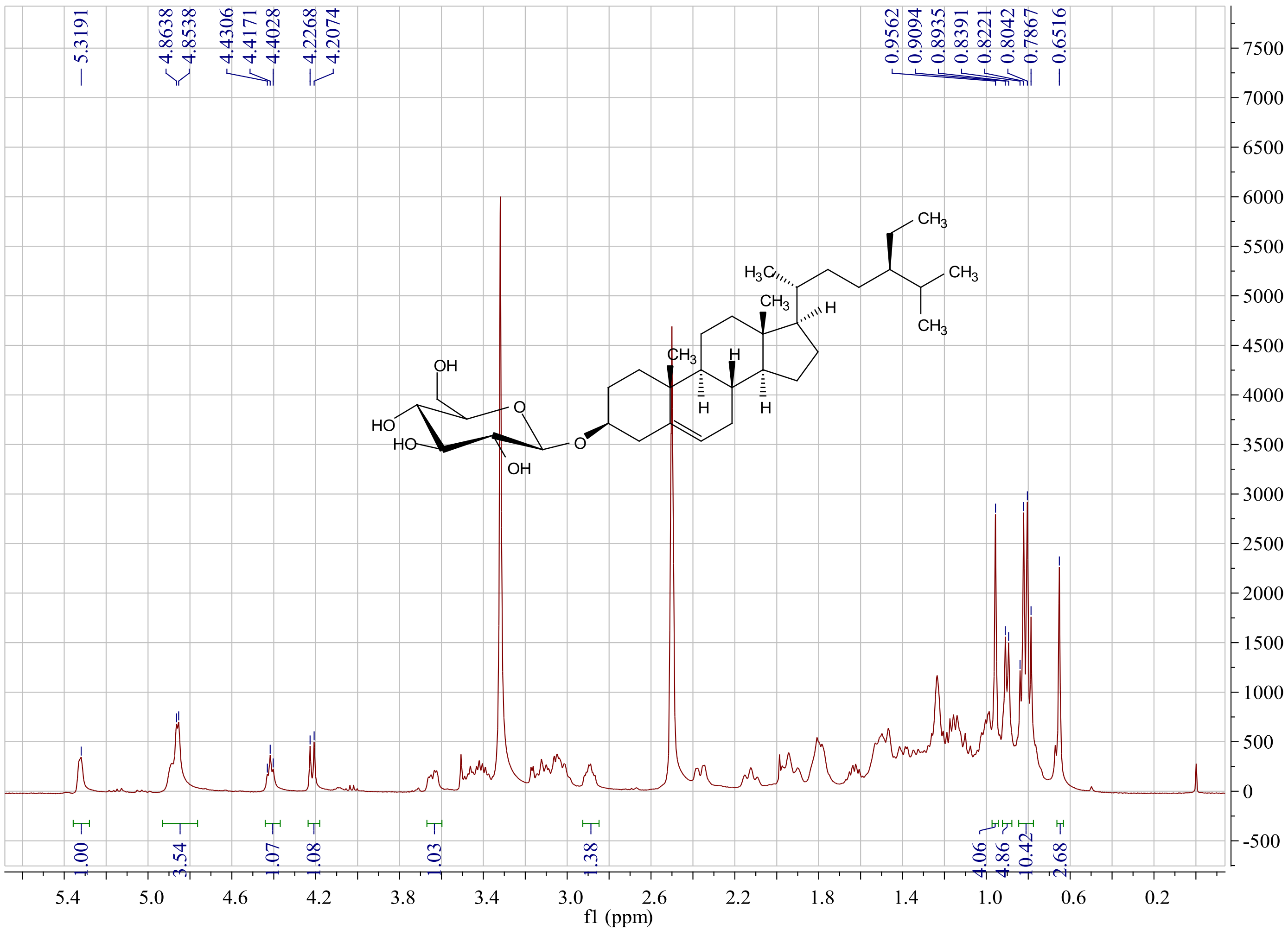

Figure S22. ^{13}C-NMR Spectrum of **5** in $CDCl_3$.

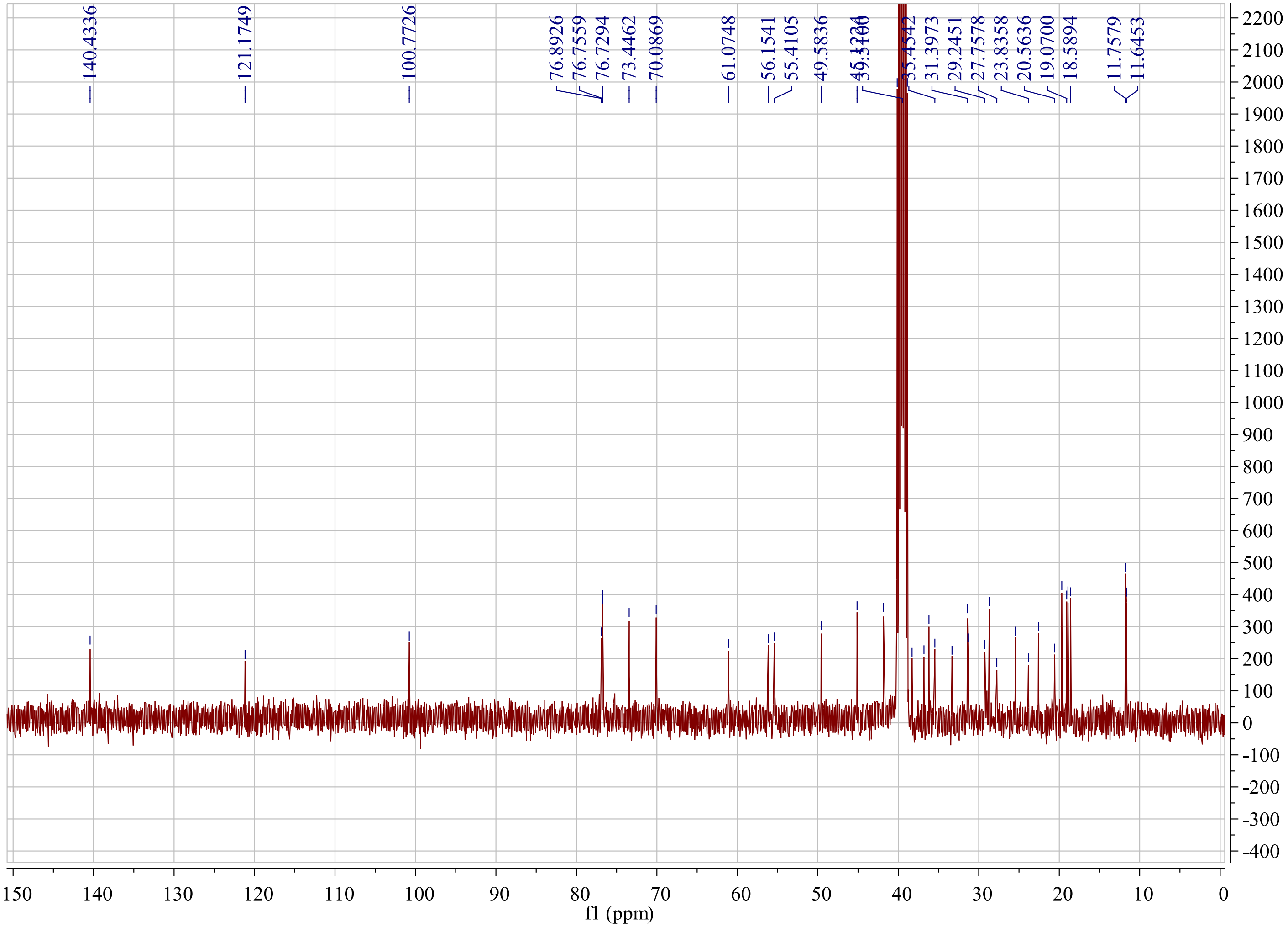

Figure S23. ^{1}H-NMR Spectrum of **6** in $CDCl_3$.

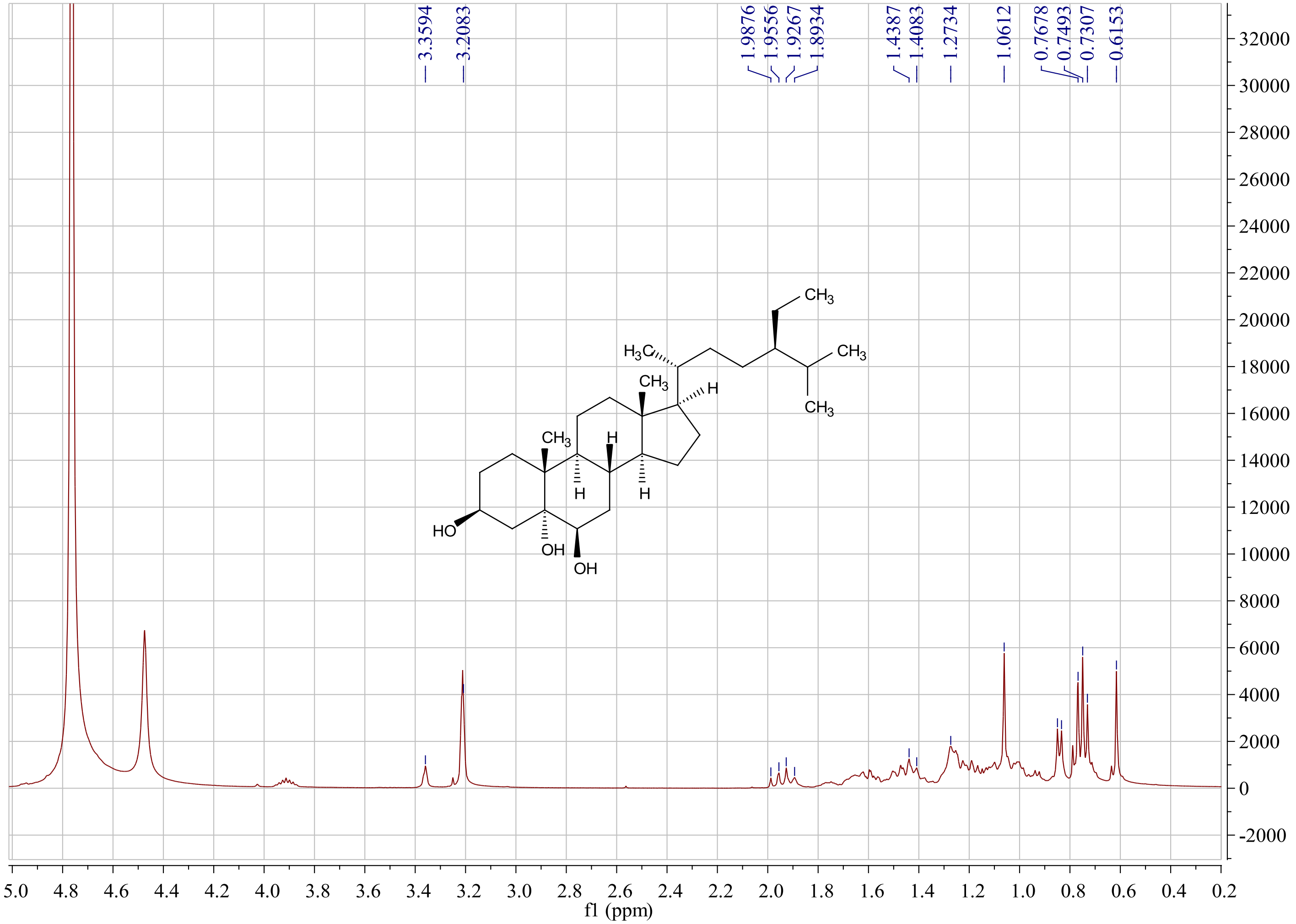

Figure S24. ^{13}C-NMR Spectrum of **6** in $CDCl_3$.

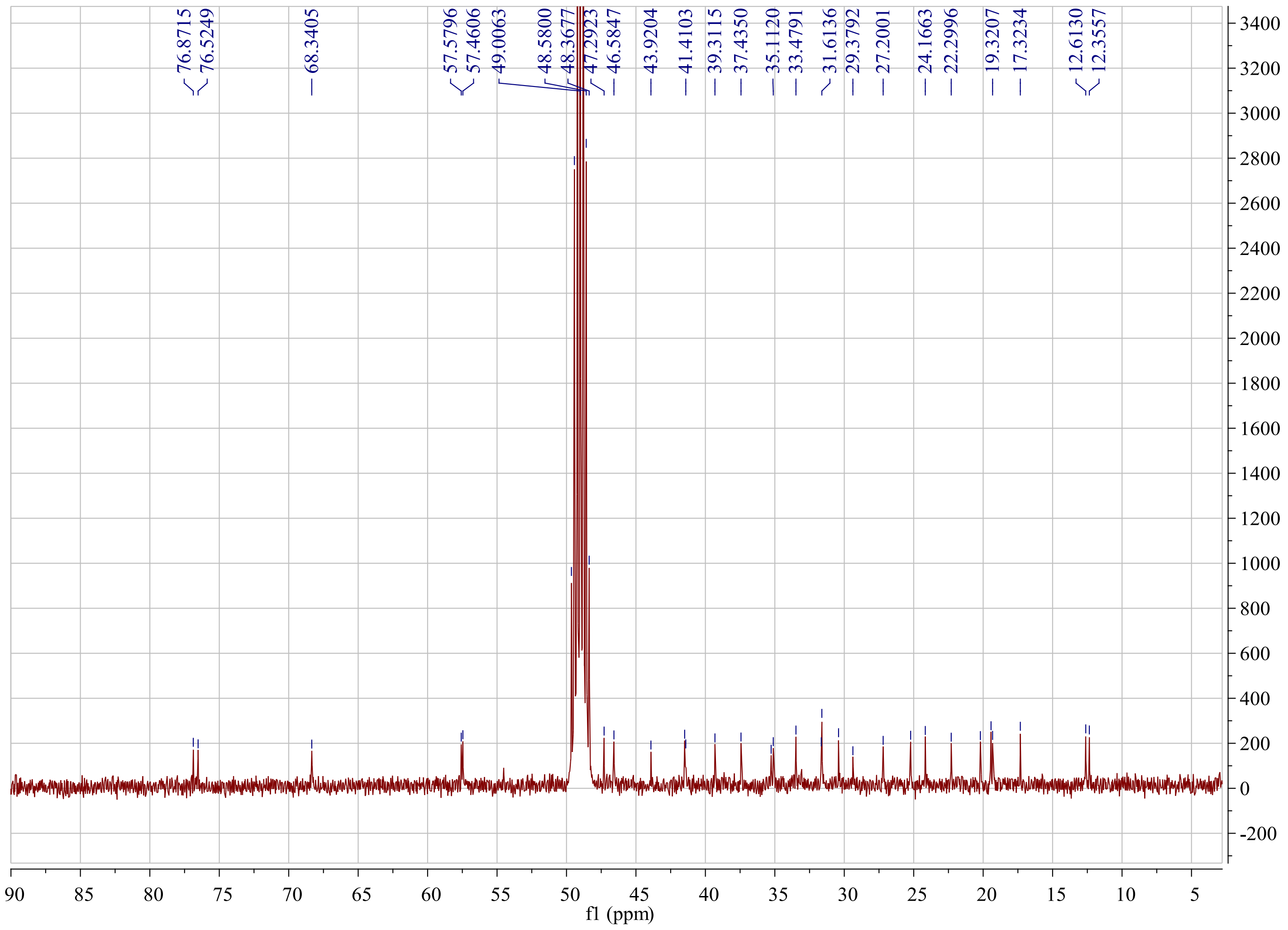

Figure S25. ^{1}H-NMR Spectrum of **7** in $CDCl_3$.

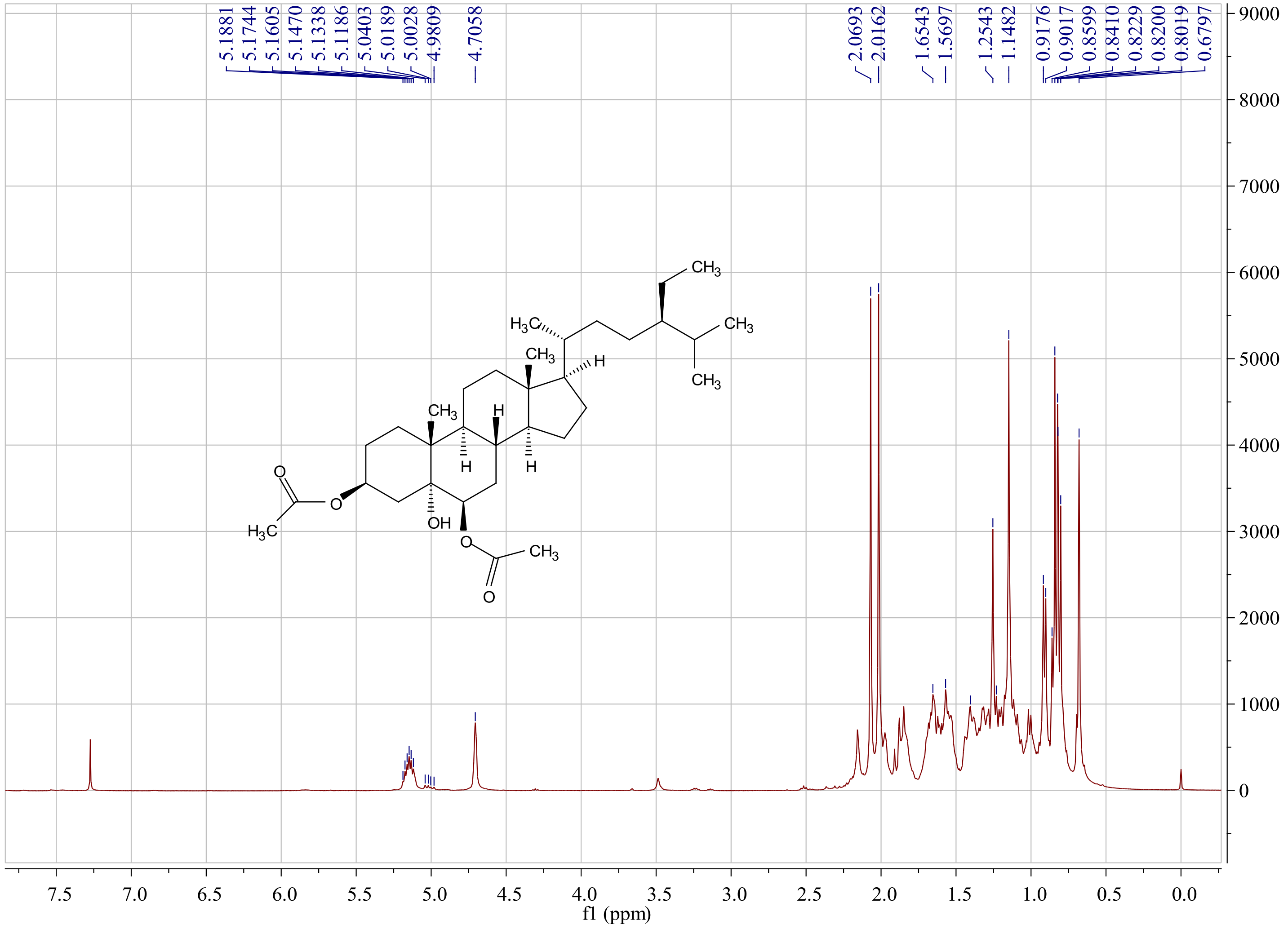

Figure S26. ^{13}C-NMR Spectrum of **7** in $CDCl_3$.

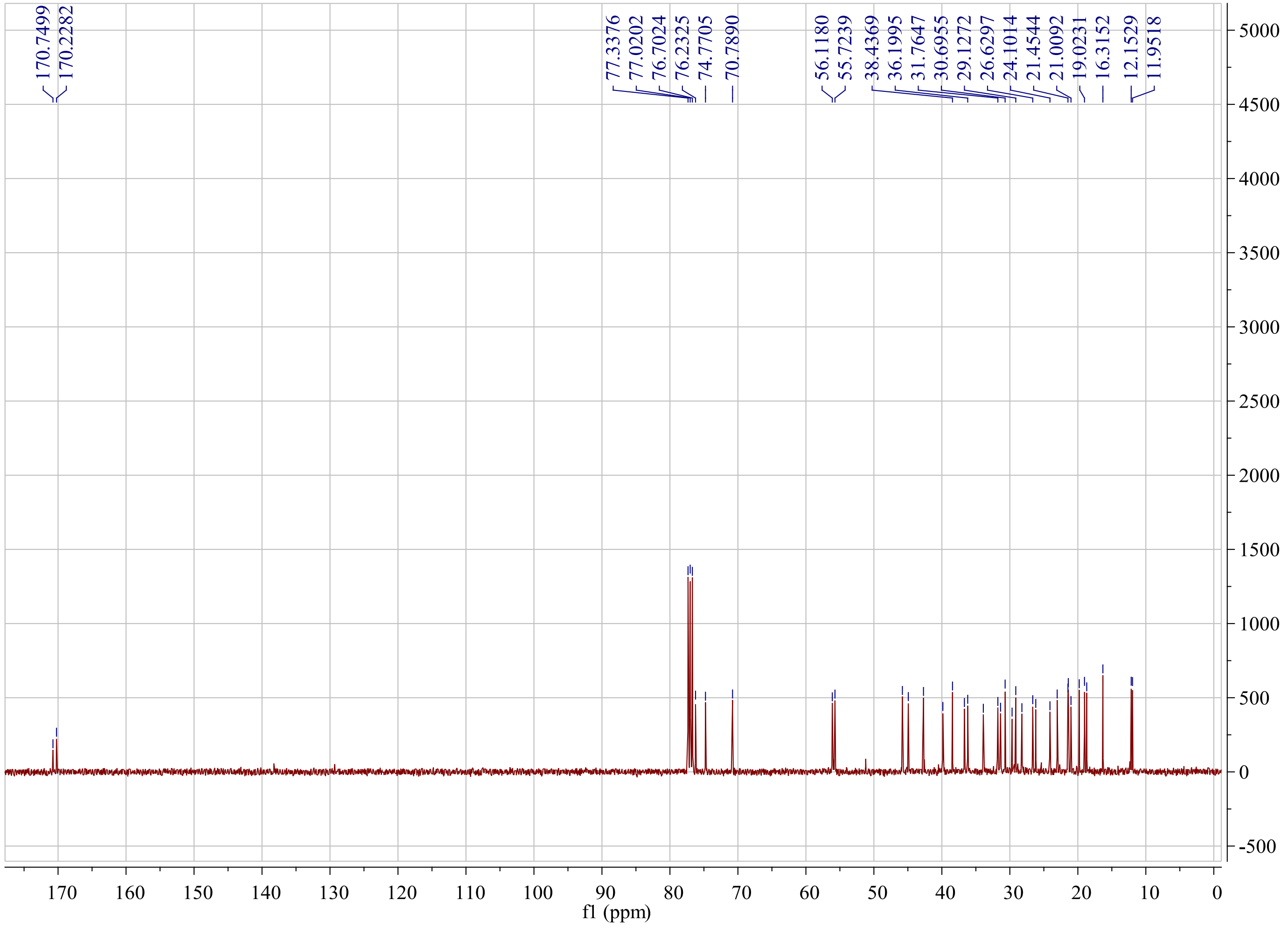

Figure S27. The selected HMBC (H ⟶ C), H-H COSY (H **—** H) and NOESY (H ⤺ H) correlations.

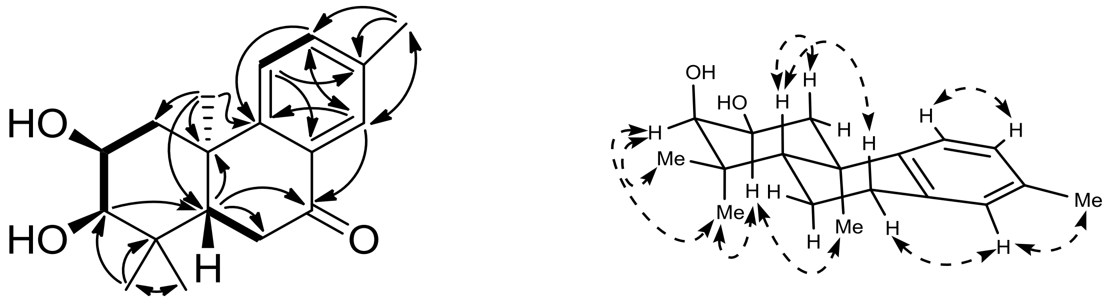

Figure S28. The CD spectra of compounds **3** and **3c**.

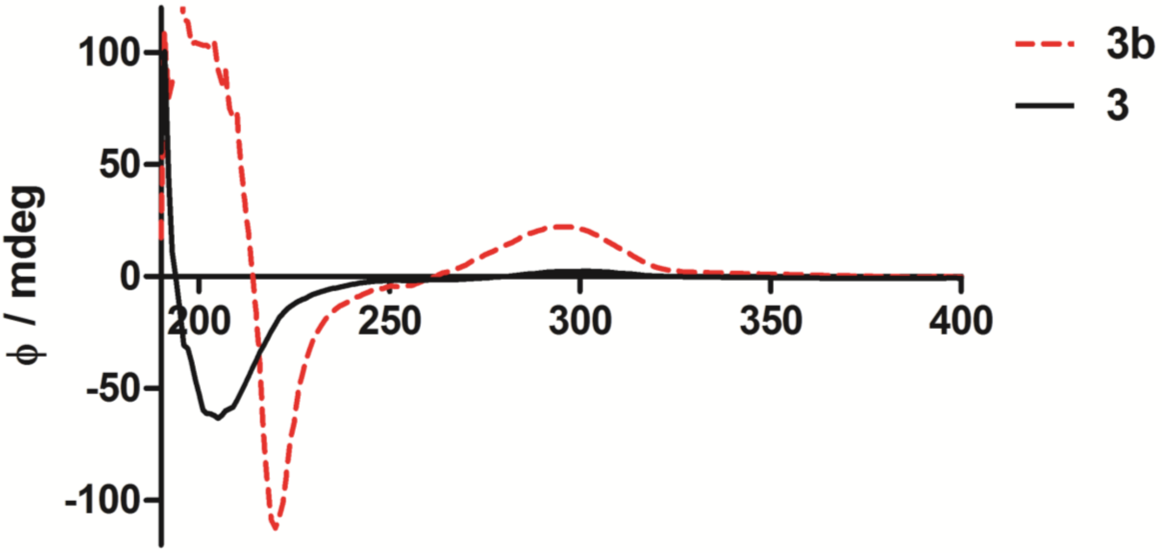

Figure S29. The entire HMBC correlations and ^{1}H-^{1}H COSY (H**—**H) correlations of compound **1**.

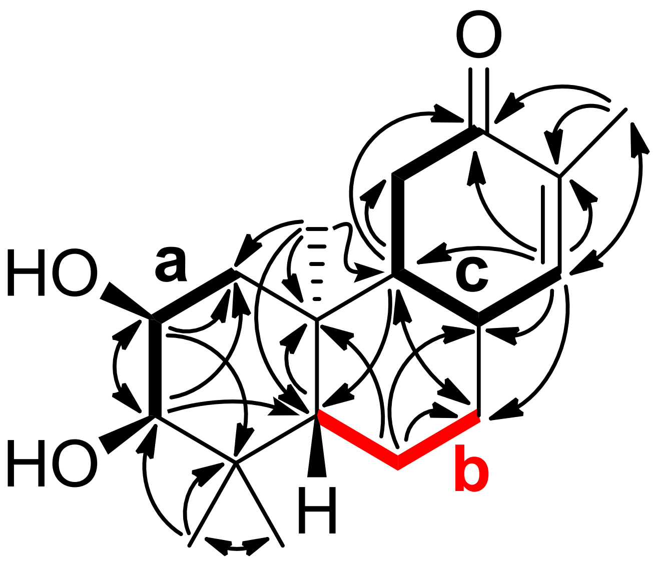

Figure S30. The selected NOESY (H↙⌒↘H) correlations of compound **3**.

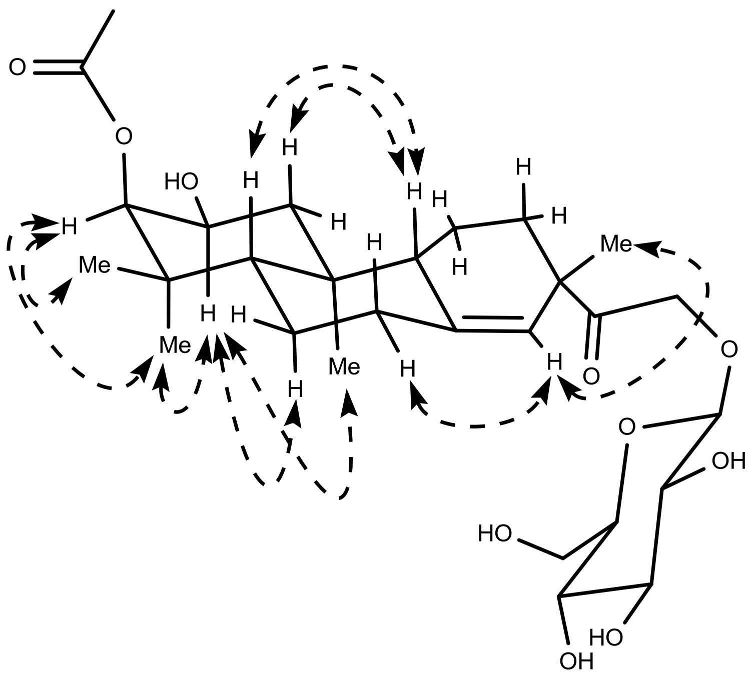

Table S1. The purities of compounds **1**–**7**.

Compound No.	Purity (%)
**1**	98.92
**2**	99.30
**3**	99.01
**4**	98.87
**5**	99.08
**6**	99.21
**7**	98.99
